# Employing deep mutational scanning in the *Escherichia coli* periplasm to decode the thermodynamic landscape for amyloid formation

**DOI:** 10.1073/pnas.2516165122

**Published:** 2025-09-17

**Authors:** Conor E. McKay, Miles Deans, Jack Connor, Janet C. Saunders, Christopher Lloyd, Sheena E. Radford, David J. Brockwell

**Affiliations:** ^a^Astbury Centre for Structural and Molecular Biology, University of Leeds, Leeds LS2 9JT, United Kingdom; ^b^School of Molecular and Cellular Biology, Faculty of Biological Sciences, University of Leeds, Leeds LS2 9JT, United Kingdom; ^c^The Discovery Centre, AstraZeneca, Cambridge CB2 0AA, United Kingdom

**Keywords:** deep mutational scanning, amyloid, Aβ_42_, machine learning

## Abstract

Deep mutational scanning (DMS) assays provide a powerful method to generate large-scale datasets essential for advancing AI-driven predictions in biology. The tripartite β-lactamase assay (TPBLA), in which a protein of interest is inserted between two domains of β-lactamase, has previously been reported as capable of detecting and quantitating the aggregation of proteins and biologics in the oxidizing periplasm of *Escherichia coli* and used as a platform for identifying small molecule inhibitors of aggregation. Here, we repurpose the TPBLA into a high-throughput DMS platform. We validate this format using a single-site saturation library of the intrinsically disordered peptide Aβ_42_, linked to Alzheimer’s disease, demonstrating strong agreement between observed variant fitness scores and variant behavior using our previously reported low-throughput TPBLA. The results of DMS revealed variant fitness scores that correlate with known amyloid-promoting regions. An in silico approach using FoldX-derived per-residue thermodynamic stability confirmed that the TPBLA reports on amyloid fibril stability. In vitro experiments support this finding, showing a strong correlation between variant fitness scores and the critical concentration of amyloid formation. Machine learning using the DMS dataset identified β‐sheet propensity and polarity as primary drivers of variant fitness scores. The derived model is also able to predict thermodynamically stabilizing regions in other amyloid systems, underscoring its generalizability. Collectively, our results demonstrate the TPBLA as a versatile platform for generating robust datasets to advance predictive modeling and to inform the design of aggregation‐resistant proteins.

## Significance

Here, we adapt the tripartite β-lactamase assay (TPBLA) performed in the oxidizing environment of the *Escherichia coli* periplasm to a high-throughput deep mutational scanning platform, expanding variant assessment to potentially many thousands at a time, facilitating comprehensive mapping of protein mutational landscapes. We apply the TPBLA to Aβ_42_ aggregation and show that its readout is the stability of the amyloid fibrils formed, which we validate by complementary in silico, in vitro, and machine-learning analyses. As the TPBLA generates high-quality, large datasets suitable as training for predictive modeling, it can guide the design of aggregation-resistant proteins and rationalize aggregation phenomena in a wide range of protein systems and be used in the evaluation of genotype–phenotype relationships in response to aggregation modulators.

Major advances in biology increasingly depend on the combination of machine learning techniques and large, high-quality datasets. DeepMind’s AlphaFold was recognized by the Nobel Prize for Chemistry in 2024 for its ability to predict protein structures from sequence alone, but its success not only rests on innovative algorithms but also relies on decades of experimental work that filled the Protein Data Bank with atomically accurate and diverse structures (1–3). Similarly, large language models such as Meta AI’s ESM-3 derive their predictive power from comprehensive sequence repositories such as UniRef, since their capacity to infer protein function, stability, and interactions depends entirely on the breadth and quality of the underlying sequence data (4, 5). As AI continues to expand into more fields of biological inquiry, acquiring ever more diverse and well-curated biological datasets remains essential for success.

Deep mutational scanning (DMS) provides a scalable route to generate large, high-quality functional datasets once the basis of selection is validated. By combining diverse mutant libraries with high-throughput screening and deep sequencing, DMS quantifies the effect of amino acid substitutions in parallel (6–8). The falling costs of next-generation sequencing (NGS) have democratized this approach, while emerging third-generation long-read technologies now enable interrogation of extended DNA regions at scale, and the parallel drop in DNA synthesis costs has made large-scale custom DNA assembly increasingly accessible (9). Together, these advances enable production of rich genotype/phenotype maps that empower machine learning models and pave the way for the next wave of biological discoveries (10).

**Fig. 1.**
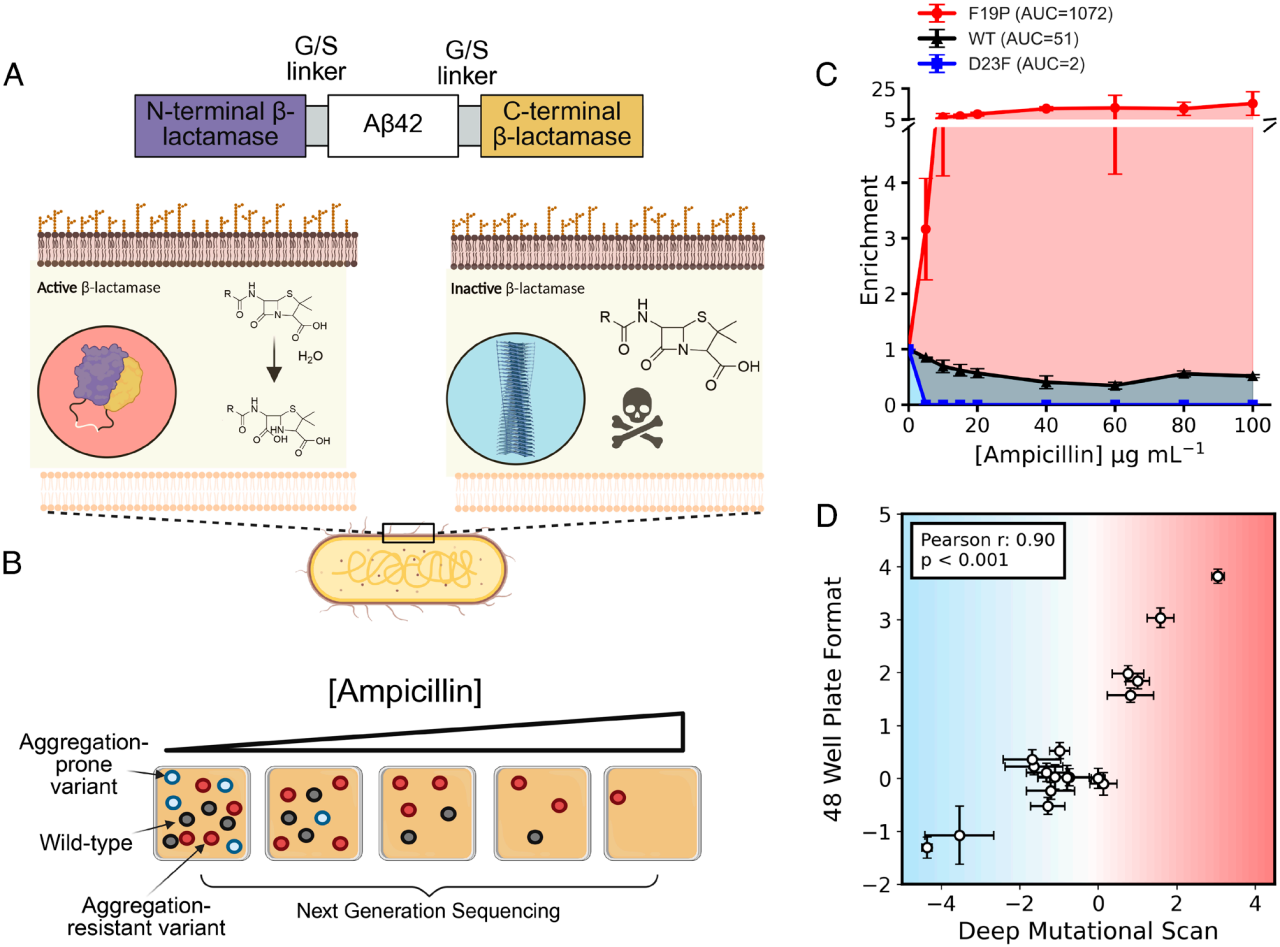
The tripartite β-lactamase assay. (*A*) The POI (in this case Aβ_42_) is flanked by the N- and C-terminal domains of TEM-1 β-lactamase, separated by a 28-residue Gly/Ser linker, and is expressed in the periplasm of *E. coli*. An aggregation-resistant POI permits the folding and function of β-lactamase, allowing hydrolysis of the β-lactam ring of β-lactam antibiotics and enabling bacterial growth under ampicillin selection. Conversely, a POI that is unable to fold and/or is aggregation-prone depletes functional β-lactamase, leading to bacteria sensitive to antibiotic. (*B*) The TPBLA in DMS format is depicted, showing an example of an aggregation-prone variant (blue), wild type (gray), and an aggregation-resistant variant (red). The read depth of the aggregation-prone variant decreases with increasing ampicillin selection as bacteria become antibiotic-sensitive, while aggregation-resistant variants outcompete the wild type. Next-generation sequencing and analysis of changes in read depth allows the assignment of variant fitness. (*C*) Representative enrichment trapezia for three variants of Aβ_42_ (n = 3): aggregation-resistant F19P (red), wild type (black), and aggregation-prone D23F (blue). Enrichment is plotted versus ampicillin concentration. The shaded trapezoidal area under each curve is integrated to generate a variant-specific aggregation score (AUC), which is then divided by the score for wild type to give a variant fitness score relative to wild type (see also *SI Appendix*, Figs. S1–S3). (*D*) A significant correlation exists between the TPBLA using the first-generation 48-well format [log_2_(AUC_Variant_)/(AUC_WT_)] and the results obtained using the assay in the third-generation DMS format (*Materials and Methods*) using 18 variants of Aβ_42_ which span a broad range of the variant fitness score observed in the deep mutational scan (R = 0.9, *P* < 0.05).

In this work, we repurpose the tripartite β-lactamase assay (TPBLA) into a DMS platform (Fig. 1 *A* and *B*). The TPBLA operates in vivo in the *Escherichia coli* periplasm, potentially offering advantages over other established DMS assays that are performed in the yeast cytosol (11). One such advantage of the TPBLA is that the *E. coli* periplasm is oxidizing, permitting analysis of disulfide-bonded proteins, including therapeutic antibody fragments (11). In addition, the periplasm of *E. coli* is permeable to small molecules, opening the door to characterizing the genotypic landscape in response to small molecule modulators of protein behavior (12). The TPBLA involves expressing a protein of interest (POI) inserted between two domains of TEM1 β-lactamase via a 28-residue G/S linker, thereby linking features of the POI (stability/solubility/aggregation propensity) to antibiotic resistance (Fig. 1*A*). Originally developed to evolve protein stability (13), the TPBLA was later adapted for screening small molecule inhibitors of amyloid formation (12), to identify key residues that drive aggregation in a model disulfide bond-containing and amyloid-forming protein (β_2_-microglobulin) (14), and to evolve developable biopharmaceuticals (11).

The first-generation TPBLA was performed in a 48-well plate (*SI Appendix*, Fig. S1), providing quantitative data (11–13). However, its low-throughput limits its suitability to generate large deep mutational datasets. In a second-generation iteration, the assay was developed as a directed evolution screen, screening variants on large agar plates and using Sanger sequencing to identify beneficial variants of β_2_-microglobulin and single-chain antibody fragments (scFvs) (11, 14) (*SI Appendix*, Fig. S2). However, in this format differences between variants cannot be quantified without subsequent analysis of individual sequences by the first-generation TPBLA format, rendering it unsuitable for machine learning. Here, we describe a third-generation of the TPBLA as an effective DMS platform, which is both high-throughput and quantitative, and show how it can be used to generate datasets of mutational landscapes conducive to machine learning (Fig. 1 *A* and *B*).

Previous work using the 48-well plate TPBLA format showed that the 42-residue peptide, Aβ_42_ associated with Alzheimer’s disease, severely compromises β-lactamase function in the TPBLA, and is more deleterious than its less amyloidogenic counterpart, Aβ_40_ (12). This demonstrates that the TPBLA can be used as a readout of the amyloid-forming properties of individual sequences, and hence, we here focus on using Aβ_42_ as the POI in a TPBLA-driven deep mutational scan. To this end, a site-saturation library of Aβ_42_ (799 potential variants including wild type) was introduced as the POI into the TPBLA and subjected to selection at increasing concentrations of ampicillin followed by NGS. Amyloid deposits upon expression of β-lactamase-Aβ_42_ (βla-Aβ_42_) were confirmed via ProteoStat staining in *E. coli*. We then used a machine learning model to identify the features driving amyloid formation and show that these features are generalizable to other amyloidogenic intrinsically disordered proteins (IDPs). The results demonstrate that the TPBLA can be used to characterize the mutational landscape of proteins in the specific biological environment of the *E. coli* periplasm for quantifying sequence-related protein behavior.

## Results

### Translating the TPBLA into a Deep Mutational Scan Format.

The aim of this study was to develop the TPBLA into an assay able to generate high-quality datasets allowing the labeling of hundreds of genotypes with quantified, clearly defined, phenotypic effects. To achieve this, we exploited the synergy between previous iterations of the assay, which at their heart is a screen able to assess a broad range of protein quality attributes manifesting in a decrease in β-lactamase activity (Fig. 1*A*). Accordingly, in a method akin to directed evolution, large variant libraries were subjected to increasing selective pressure by growth on solid medium containing increasing concentrations of ampicillin (Fig. 1*B*). In contrast to traditional methods of directed evolution (15), the phenotypic effect of advantageous and deleterious substitutions can be tracked and quantified by NGS by measuring the change in abundance of each variant gene upon growth at increasing selective pressure. Plotting relative enrichment of each variant versus the ampicillin concentration generates a “survival curve” (Fig. 1*C*) similar to those obtained using the 48-well TPBLA format (*SI Appendix*, Fig. S1*B*) (11–14). Finally, a single relative fitness score is calculated as the (log_2_) ratio of the area under the curves (AUCs) of the variant and wild-type (WT) sequences. This approach is thus analogous to that used in DMS, with the exception that many (16–18), but not all (6, 19), DMS studies to date have been performed in liquid culture in yeast. We, and others, have shown that the TPBLA can exhibit phenotype-genotype uncoupling due to leakage of the normally periplasmic-sequestered β-lactamase into the growth culture, and hence, the selection was performed on solid agar plates (20).

To verify that the third-generation DMS TPBLA format yields results that are comparable with those using growth scores obtained with the previously deployed first-generation 48-well format (11–13), each method was used to score 18 βla-Aβ_42_ variants. These variants were selected to be assayed in the 48-well format as they spanned the range of variant fitness score observed in the TPBLA deep mutational format. The results showed that while the two different generations of the assay yield different absolute scores, their correlation is excellent (Pearson R = 0.9, *P* < 0.001) (Fig. 1*D*), validating the third-generation TPBLA for use in high-throughput analysis of genotype–phenotype relationships. Using this third-generation format, the TPBLA allows facile genetic manipulation and measurement of fitness in a single set of experiments that only require overnight incubation of bacterial growth on solid medium.

### Deep Mutational Scan of Aβ_42_.

As described above, we have previously applied the TPBLA in 48-well plate format to different amyloidogenic proteins and peptides and showed that amyloidogenic sequences such as human IAPP (hIAPP) and Aβ_42_ exhibit greater sensitivity to ampicillin relative to their less aggregation-prone variants (rat IAPP/Aβ_40_) or constructs containing a Gly-Ser linker of similar length as the POI, despite no clear difference in their expression levels (12). For example, the log_2_ of the ratio of the area of the 48-well format survival curve using a Gly-Ser-linker as the POI relative to Aβ_42_ is 5 at 0 to 140 µg/mL ampicillin (12). Given the strong link between fitness and amyloid propensity in the TPBLA, we performed a deep mutational scan of Aβ_42_ to determine the effect of all possible single-residue amino acid substitutions on fitness (*Materials and Methods*). Accordingly, a commercially sourced *E. coli* codon-optimized site-saturation library, comprising single substitutions of every canonical amino acid at every residue position was cloned into the previously described pBR322-βla plasmid adapted for Golden Gate cloning (*Materials and Methods*) (11). The resulting variant library (βla-Aβ_42lib_) was transformed using electroporation into TG1 cells and DNA was extracted (*Materials and Methods*). For the deep mutational scan, commercial *E. coli* SCS1 cells were transformed with βla-Aβ_42lib_ and grown in liquid culture for 2 h followed by further incubation with 0.075% (w/v) arabinose to induce expression of βla-Aβ_42lib_. After spreading onto a series of LB agar plates in the presence of 0 to 100 µg mL^−1^ ampicillin and 0.075% (w/v) arabinose and incubation for 18 h, the plasmid DNA from each plate was harvested and sent for Azenta Amplicon-EZ NGS. After genotype analysis of the sequencing results, the variant fitness scores relative to WT Aβ_42-_were quantified (*Materials and Methods* and *SI Appendix*, Fig. S3).

**Fig. 2.**
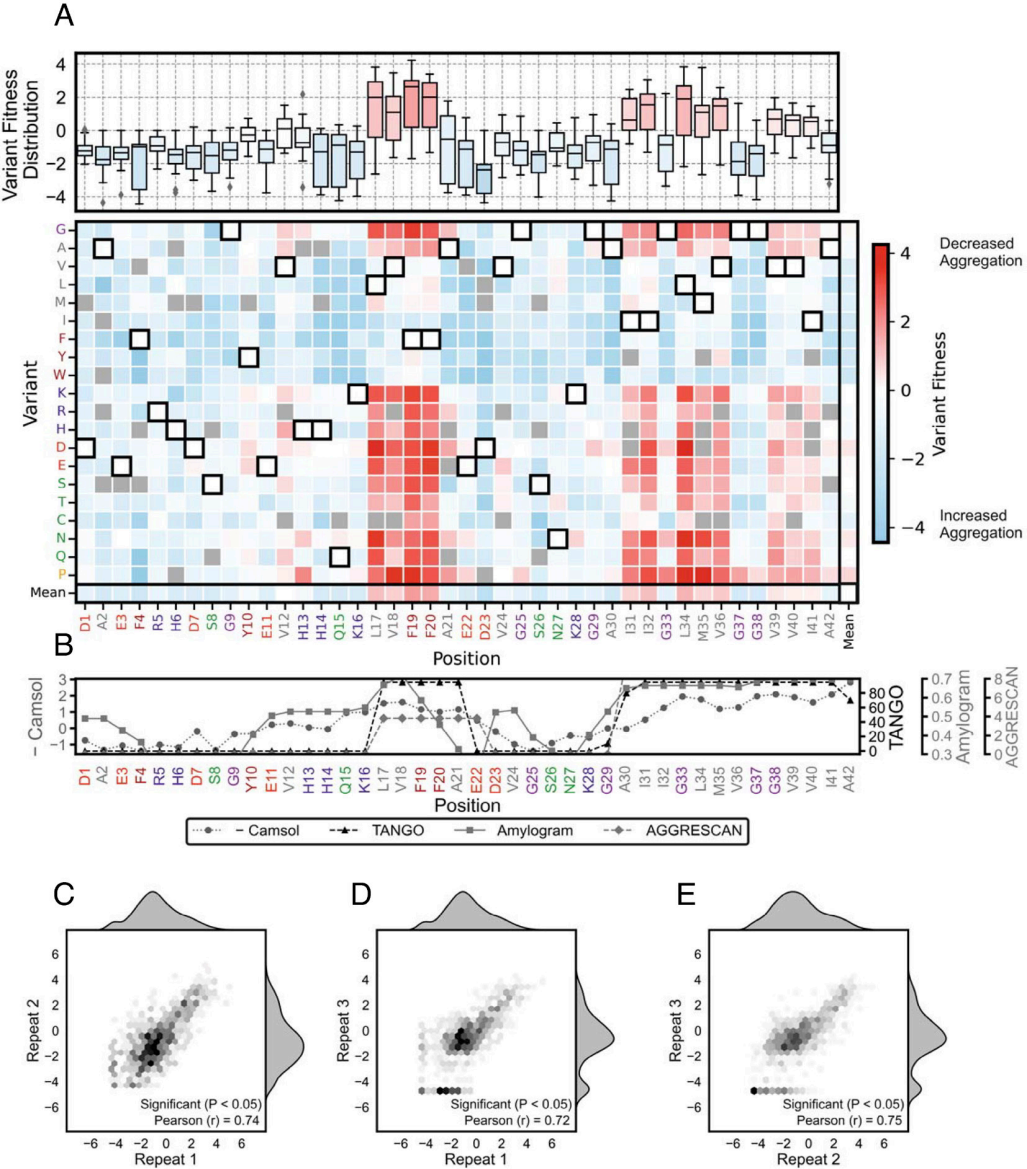
Deep mutational scan of Aβ_42_ using the TPBLA. (*A*) (*Upper*) Box-and-whisker plots of variant fitness scores at each Aβ_42_ residue: the central line denotes the median, the box spans the interquartile range (IQR), and whiskers extend 1.5 × IQR. (*Lower*) Heatmap of variant fitness scores for single amino acid substitutions obtained using the TPBLA selection. The horizontal axis displays the WT primary amino acid sequence. The vertical axis represents the variant amino acid introduced. WT amino acids are highlighted with a black box. Missing variants are colored gray. The *Bottom* row and rightmost column display the average for that row/column. (*B*) Aggregation propensity profiles for Aβ_42_ predicted by the algorithms Camsol (23), TANGO (24), AmyloGram (25), and AGGRESCAN (26). The Camsol scores have been inverted (× –1) so that, like the others, higher values reflect increased aggregation propensity. Algorithms predict solubility (–Camsol), β-aggregation propensity (TANGO), self-assembly probability (AmyloGram), and overall aggregation propensity (AGGRESCAN). (*C*–*E*) Correlation between variant fitness scores obtained from (*C*) repeats 1 and 2, (*D*) repeats 1 and 3, and (*E*) repeats 2 and 3.

The heatmap of variant fitness scores derived from βla-Aβ_42lib_ is shown in Fig. 2*A*. Of the 798 possible single-point substitutions, 749 (94%) were present in our library. Importantly, we observed no positions where an entire type of amino acid class was missing. The average number of reads a variant achieved in the unselected libraries was 39, with read depth increasing as a function of ampicillin pressure. This far exceeds the average signal threshold of 3, set by the misread rate in the invariant Gly-Ser linker upstream to Aβ_42_ in each NGS reaction (*Materials and Methods*). Regions in Aβ_42_ were observed in which most substitutions are beneficial (residues L17-F20 and I31-I41), which align with the regions known to promote aggregation from other studies (21, 22) and are predicted by Camsol (23), TANGO (24), Amylogram (25), and AGGRESCAN (26) (Fig. 2*B*). The reproducibility of variant fitness scores was confirmed by correlation between biological repeats (Repeat 1/Repeat 2 Pearson R = 0.74, *P* < 0.05; Repeat 1/Repeat 3 Pearson R = 0.72, *P* < 0.05; Repeat 2/Repeat 3 Pearson R = 0.75, *P* < 0.05) (Fig. 2 *C*–*E*). Both increases in fitness (shaded red, assumed to reflect decreased aggregation) and decreases in fitness score (shaded blue, presumably increased aggregation) were observed. Variant fitness scores ranged from −4.44 (F4W) to 4.24 (F19P). Among the substitutions, 16% increased the fitness score by at least 1, 45% decreased the fitness score by 1 or more, and 39% resulted in a change in fitness score between −1 and 1. The distribution of fitness scores are centered on a value of −0.87 (*SI Appendix*, Fig. S4*A*). In general, variant fitness scores increased positively with introduction of a less hydrophobic residue (introduction of Arg/Lys/Asp/Glu/Asn/Gln/His/Ser), or with substitution with proline or glycine, consistent with aggregation being the driver of fitness in the selections (*SI Appendix*, Fig. S4*B*).

The selection also showed that introduction of amino acids at different positions causes distinct phenotypic responses (Fig. 2*A*). Clear patterns are evident, especially when residues are grouped by their similarity in response to substitution by each of the canonical amino acids (*SI Appendix*, Fig. S5). There are two “stripes” of residues evident in Fig. 2*A* in which substitution with charged or polar residues, glycine, or proline increases fitness, comprising residues L17-F20 and I31-I41, which correspond to the well-known aggregation-prone regions (APRs) of Aβ_42_ (21, 22). Interestingly, Gly33, Gly37, and Gly38 are notable exceptions, with substitution of these residues generally decreasing fitness, possibly because of the introduction of an amino acid with a larger side chain promoting aggregation. Previous results have shown that substitution of Gly33 with Ala or Ile increases higher-order oligomer formation, as measured by size-exclusion chromatography (27), which could contribute to the decreased fitness of these variants. Substitution of residues in the two APRs of Aβ_42_ with charged residues increases fitness, with introduction of negatively charged residues having a more pronounced effect than substitution with positively charged residues, consistent with the stronger “gatekeeper” properties of negatively charged residues (28). The effect of substitutions in residues L17-A21 (mean variant fitness score 1.43, SEM = 0.20) is stronger than that for residues in the second APR (I32-I41) (mean variant fitness score = 0.76, SEM = 0.11). Two other notable residue responses are observed: For D23, all substitutions decrease variant fitness. For F4 and S26, substitution also generally decreases variant fitness (note also that substitutions of F4 yield responses distinct from the other two phenylalanine resides in the sequence (F19 and F20)). This observation highlights that the TPBLA captures position-specific interactions dependent on the position of the amino acid. Finally, substitution with bulky hydrophobic residues (Trp, Tyr, Phe, Leu, Ile, Val, Met, and Cys) generally causes a pronounced fitness decline. Interestingly, substitutions with Thr generally decrease variant fitness at different positions in the sequence (mean = −0.50, SEM = 0.21) compared to substitutions with Ser, Asn, or Gln, which broadly increase variant fitness (mean = 0.08, SEM = 0.15), consistent with the β-branched Thr side-chain enhancing amyloid formation (29). Notably, the 22 known familial mutations in the coding region of Aβ_42_ give variant fitness scores ranging from −1.69 (E22Q Dutch mutation) to 1.16 (A21G Flemish mutation) (*SI Appendix*, Table S1). This range of responses could reflect the variety of biological processes that contribute to Alzheimer’s disease, including APP processing, aggregation rate, and the ratio of Aβ_42_ to Aβ_40_ sequences (30). Together, these observations support the notion that the phenotypic trait being quantified by the TPBLA screen is related to amyloid formation in the periplasm of *E. coli* after overexpression of βla-Aβ_42_.

### Amyloid Formation in the *E. coli* Periplasm.

**Fig. 3.**
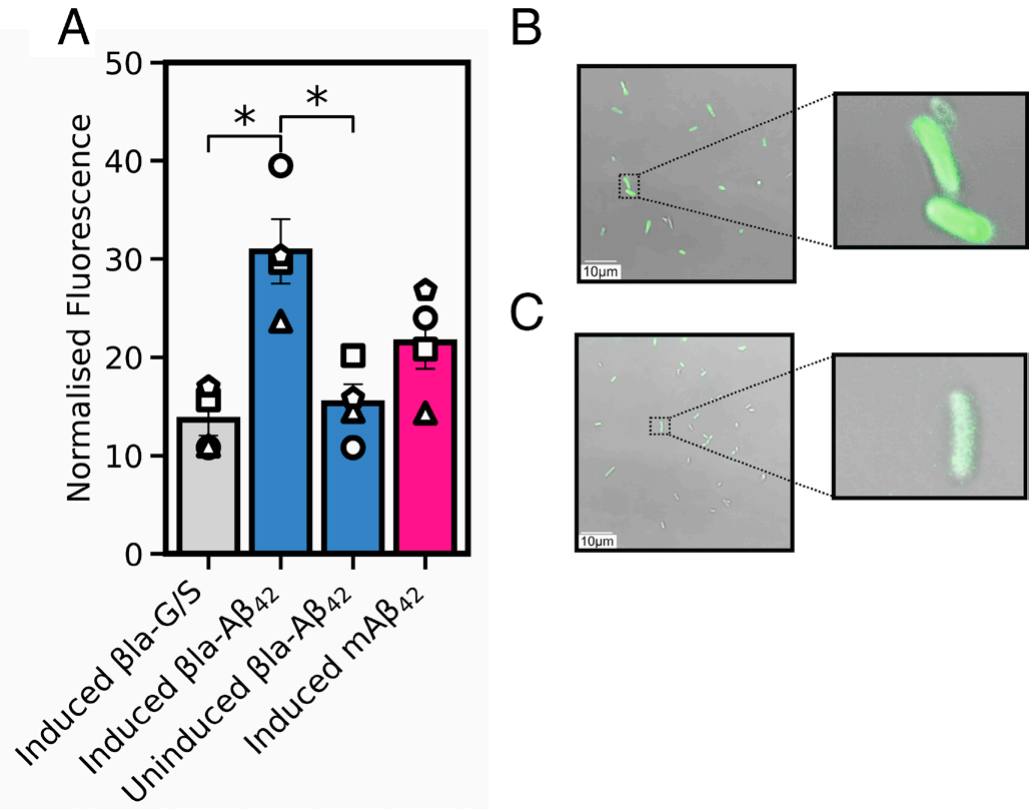
Protesostat fluorescence imaging of bacterial cells expressing mAβ_42_, βla-G/S, βla-Aβ_42_, or βla-Aβ_42_ (uninduced). (*A*) Normalized fluorescence intensity: βla-Aβ_42_ exhibited significantly higher fluorescence than uninduced cells (lacking 0.075% arabinose induction) or βla-G/S. Statistical significance was determined using an unpaired *t* test between βla-G/S and βla-Aβ_42_, and a paired *t* test between uninduced βla-Aβ_42_ and βla-Aβ_42_, both of which were significant (*P* < 0.05). (*B* and *C*) Confocal fluorescence images of (*B*) bacteria expressing βla-Aβ_42_ and (*C*) uninduced βla-Aβ_42_, each visualized with 488 nm excitation and a 500 to 600 nm filter for detection (*Materials and Methods*).

To investigate whether βla-Aβ_42_ forms amyloid deposits in the *E. coli* periplasm, bacteria expressing βla-Aβ_42_ grown overnight on solid medium were resuspended in liquid culture and incubated for 30 min with Proteostat dye (*Materials and Methods*). This dye has been shown to yield enhanced fluorescence in the presence of preformed Aβ_42_ amyloid fibrils in vitro and in the *E. coli* cytoplasm following overexpression of Aβ_42_ containing an N-terminal methionine (mAβ_42_) (31). Bacteria expressing WT βla-Aβ_42_ showed a 2.0-fold higher fluorescence signal compared to uninduced cells (*P* < 0.05) and a 2.3-fold higher signal than those expressing a 28-residue Gly-Ser linker (βla-G/S) instead of βla-Aβ_42_ (*P* < 0.05) (Fig. 3*A*). The fluorescence emission intensity for βla-Aβ_42_ is also ~1.5-fold higher than bacteria expressing mAβ_42_ which forms intracellular inclusion bodies (32) (Fig. 3*A*). Confocal microscopy also showed that bacteria expressing wild‐type βla‐Aβ_42_ exhibited more fluorescence than uninduced cells in the presence of Proteostat dye (Fig. 3 *B* and *C*). These observations are consistent with amyloid formation in *E. coli* upon the expression of βla-Aβ_42_.

### The TPBLA Screens for Amyloid Stability.

Two studies have previously reported deep mutational scans on Aβ_42_ (33, 34) using yeast-based reporters as opposed to the TPBLA which takes place in the periplasm of *E. coli.* In the yeast-based assays, Aβ_42_ variants are assayed in the cytosol by fusing Aβ_42_ to the N terminus of dihydrofolate reductase (Aβ_42_-DHFR) (33) or to the C-terminus of Sup35N, the nucleation domain of Sup35 (Sup35N-Aβ_42_) (16, 34). In the DHFR system, Aβ_42_ aggregation sequesters the complex into aggregates, impairing the ability of the enzyme to fold and function. Addition of methotrexate (a competitive DHFR inhibitor) further amplifies selection pressure so that only cells expressing aggregation-resistant Aβ_42_-DHFR fusions can maintain enough DHFR activity to grow, allowing survival to serve as a proxy for aggregation propensity (33) (*SI Appendix*, Fig. S6*A*). In the Sup35N-Aβ_42_ system, Sup35N-Aβ_42_ aggregation seeds aggregation of endogenous prion domain of Sup35 (Sup35p) enabling read-through of premature stop codons and permitting growth in an adenine-deficient medium (*SI Appendix*, Fig. S6*B*) (34). This acts as a positive selection for Aβ_42_ amyloid formation, contrasting with the negative selection in the Aβ_42_-DHFR assay. For Sup35N-Aβ_42_, the authors reported that fitness scores (referred to as “nucleation scores”) correlated with previously published nucleation rates of fibril formation of a selection of Aβ_42_ variants monitored by Thioflavin-T (ThioT) assays in vitro (16). The results of the TPBLA DMS dataset strongly correlate with those obtained using Aβ_42_-DHFR (Pearson R = 0.79, *P* < 0.05, *SI Appendix*, Figs. S6*A* and S7*A*). The authors of the Aβ_42_ -DHFR study concluded that the two regions with high solubility (fitness) scores were “regions are most likely to form buried β-stands.” By contrast, the TPBLA data for βla-Aβ_42_ only weakly correlate with those obtained using Sup35N-Aβ_42_ (Pearson R = −0.1, *P* < 0.05) (*SI Appendix*, Figs. S6*B* and S7*B*). The differences in assay readouts emphasize the value of using different screens to assess a protein’s properties and highlight that different screens may output different results dependent on the trait that is most sensitive under the assay conditions used. Despite these differences, the results highlight that the TPBLA DMS provides an orthologous screen for the sequence dependence of the amyloid propensity of Aβ_42_ in a different biological setting.

**Fig. 4.**
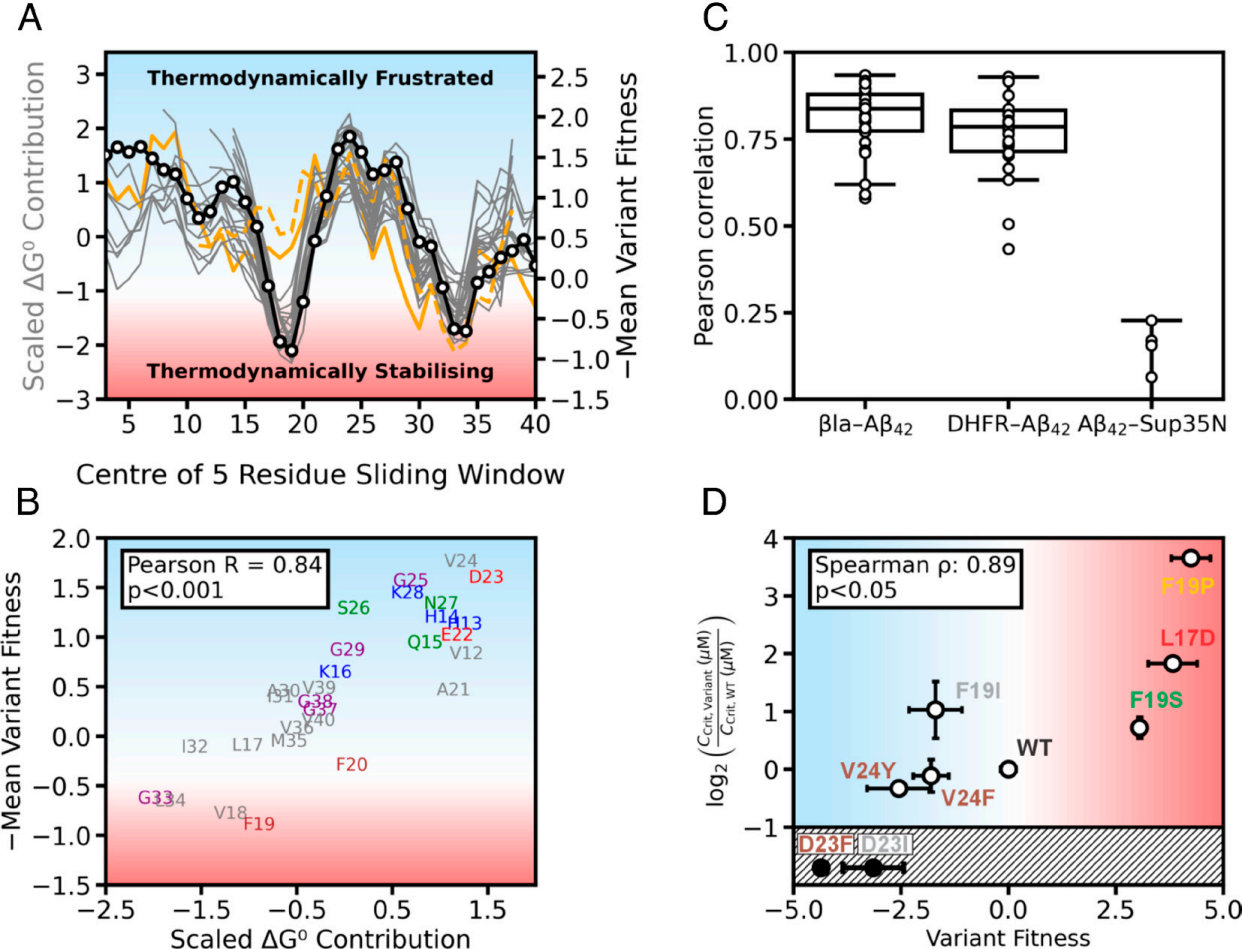
The readout of the TPBLA is the thermodynamic stability of the amyloid fibril fold: (*A*) A strong correlation exists between residues important for stability calculated by FoldX and residues that, when substituted, lead to a significant change in TPBLA variant fitness score. Shown is an overlay of the per-residue free energy contribution for all Aβ_40_ and Aβ_42_ (gray) structures and per-position mean TPBLA variant fitness score inverted (× –1) (black bold line and datapoints), both analyzed over a five-residue sliding window. Structures which do not agree as well with TPBLA scores are shown as orange lines (PDB:8SEK (dashed), PDB:5OQV (continuous). (*B*) A strong positive correlation exists between variant fitness scores measured in the TPBLA and per-residue stability contribution. Shown is TPBLA mean variant fitness score inverted (× –1) correlated with per-residue stability contribution of PDB:8OL3 (42), which has the median Pearson value (R = 0.84) for all structures. (*C*) Distribution of Pearson correlation coefficients between the mean per residue fitness achieved by βla-Aβ_42_, Aβ_42_-DHFR, and Sup35N-Aβ_42_ with ΔG^0^ contribution per position for all Aβ_40_ and Aβ_42_ amyloid structures considered over a five-residue sliding window (n = 21). (*D*) Correlation of in vitro-derived critical concentration (C_crit_) with variant fitness scores for a panel of purified Aβ_42_ variants (D23F, D23I, V24Y, V24F, F19I, wild type, F19S, L17D, and F19P) colored by the identity of the introduced amino acid class as used in Fig. 2*A*. Variants D23F and D23I could not be purified as monomers so are in the hatched region of the phase diagram in black. A significant correlation between TPBLA variant fitness score and log_2_(C_Crit Variant_/C_Crit WT_) is observed for this panel of variants (ρ = 0.89, *P* < 0.05).

To understand the molecular origins of selection by the TPBLA screen, we exploited the recent explosion in available high-resolution Aβ_42_ amyloid structures obtained using cryoEM (35), and combined this information with calculation of the energetic contributions of each residue in the structured fibril core using the algorithm FoldX (*Materials and Methods*) (36, 37). Previous analyses have shown that the regions that stabilize (approximately 30% of residues) or destabilize the amyloid fold of different fibril polymorphs (different amyloid structures that result from the same sequence) are shared in the different amyloid folds (37, 38). This suggests that amyloid polymorphism arises from differences in intra- and interprotofilament contacts between these stabilizing regions. Residues that destabilize Aβ_42_ fibrils would thus be expected to increase the TPBLA fitness score, with those that stabilize amyloid decreasing fitness relative to wild type, irrespective of the polymorph formed in the bacterial periplasm. To test this hypothesis, we calculated the per-residue thermodynamic stability for 26 WT Aβ_40_/Aβ_42_ amyloid fibril structures currently available in the Amyloid Atlas (35) over a five-residue running average using FoldX, as described previously (36, 37). To quantify the importance of specific contacts, and thus the energetic contribution made by each residue in our DMS study, we calculated the average fitness score for all substitutions at each position, again averaged over a five-residue running average. Comparison of these profiles (Fig. 4*A*) reveals a strong positive correlation (median R = 0.84) (Fig. 4*B*). The two structures with the poorest correlation have distinct origins: Aβ_40_ fibrils extracted from the brain of a patient with Down’s syndrome [8SEK (39)], and Aβ_42_ fibrils formed under low‐pH conditions in the presence of an organic cosolvent [5OQV (40)]. Contrary to the pattern observed with the other Aβ_40_/Aβ_42_ fibrils in our dataset, these two structures are not calculated to be strongly stabilized by APR1 (L17-A21). Rendering an Aβ_42_ amyloid fibril structure solved by cryo-EM which achieved a median agreement between the FoldX-determined fibril stability and the TPBLA fitness scores shows that APR 1 (L17-A21) and APR 2 (I32-A42) form the stabilizing core, rationalizing why substitution with charged or polar residues is destabilizing (*SI Appendix*, Fig. S8). Each APR is juxtaposed with frustrated residues i.e., residues whose substitution generally increases amyloid stability (e.g., residues H14-K16 and E22-D23). Such “gatekeeper” residues have been observed previously and their mutation to a hydrophobic amino acid could extend the APR, increasing fibril stability and decreasing fitness, as was observed experimentally in the TPBLA screen (Fig. 2*A*). Notably, substitutions of the gatekeeper residues E22 and D23 have been linked to familial Alzheimer’s disease (41). As expected, based the results of the TPBLA, the Aβ_42_-DHFR DMS dataset also correlates well with the calculated per-residue stability of Aβ_42_ and Aβ_40_ fibrils (33). In contrast, the Sup35N-Aβ_42_ dataset does not (Fig. 4*C*). Taken together, these results underscore that the readout of the TPBLA (and likely also the Aβ_42_-DHFR screen) reflects the local thermodynamic stability of the endpoint amyloid fibril. In accordance with this conclusion, we observed a stronger agreement of fibril thermodynamic stability with the observed TPBLA dataset than that achieved by TANGO, AGGRESCAN, Amylogram, or Camsol, despite the inclusion of 8SEK and 5OQV in the dataset (*SI Appendix*, Fig. S9).

To validate the prediction of fibril stability using FoldX, we determined the critical concentration (C_crit_) of amyloid formation in vitro for selected Aβ_42_ variants in the absence of the β-lactamase scaffold. C_crit_ is defined as the monomer concentration at which the rates of amyloid fibril dissociation and monomer sequestration are equal (43). Given the high thermodynamic stability of amyloid fibrils and thus low monomer concentration at equilibrium, experimental verification of C_crit_ is challenging, but has been reported to range from 50 to 100 nM for WT Aβ_42_, depending on the technique or growth condition used (44, 45). To this end, we measured the fluorescence intensity of the amyloid binding dye ThioT at the end point of the amyloid growth phase across a range of initial Aβ_42_ monomer concentrations and used this as a proxy of fibril yield (46). Linear extrapolation of this value versus the initial Aβ_42_ concentration was then used to determine the minimal initial monomer concentration able to form amyloid (C_crit_) (*Materials and Methods*). Nine Aβ_42_ variants (in the absence of βla) with diverse variant fitness scores were selected for this analysis. Two variants with large negative fitness scores at the gatekeeper position of D23 (fitness scores of −3.05 and −4.17 for D23I and D23F, respectively) could not be purified as a monomer by gel filtration consistent with enhanced aggregation propensity (*Materials and Methods*) and were thus excluded from the analysis. The extrapolated C_crit_ values for the remaining seven variants plotted against variant fitness scores are shown in Fig. 4*D* and endpoint ThioT fluorescence intensity versus initial Aβ_42_ concentration is shown in *SI Appendix*, Fig. S10. We observed a significant nonlinear positive correlation between the TPBLA variant fitness scores and the determined C_crit_ (ρ = 0.89, *P* < 0.05), consistent with fibril stability being the dominant readout of the selection pressure in our screen. This nonlinearity is common in DMS assays owing to the observable output saturating at high variant fitness scores (10, 17). Finally, we extracted the macroscopic rate constants for primary and secondary events in amyloid formation [λ and κ, respectively (47)] by fitting the kinetics of amyloid formation measured by ThioT fluorescence for the 7 variants at an 8 µM initial monomer concentration (*SI Appendix*, Figs. S11 and S12). No significant correlation was observed between the TPBLA variant fitness scores and λ (ρ = 0.61, *P* > 0.05) or κ (ρ = 0.71, *P* > 0.05). This lack of significance is consistent with the thermodynamic stability of amyloid fibrils being primarily responsible for the fitness scores obtained in our assay.

### A TPBLA-Trained Random Forest Model Is Empowered by Features Known to Be Related to Amyloid Stability.

**Fig. 5.**
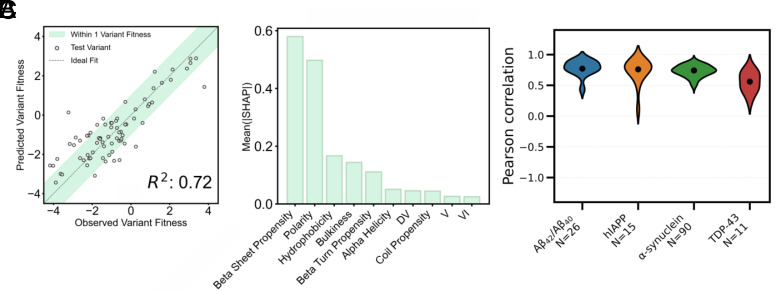
Random forest model trained on the βla-Aβ_42_ dataset (*A*) The random forest was trained using one-hot encoded amino acid identities, dipeptide composition, and additional sequence-derived features to predict βla-Aβ_42_ variant fitness scores. Performance was evaluated by 10‐fold cross‐validation. Predicted fitness scores for tested variants are plotted against observed scores, yielding a median R^2^ of 0.72 across folds. (*B*) The top 10 features used in the model are ranked by their mean absolute SHAP values. Higher SHAP values indicate a greater impact on the model’s predictions. (*C*) Violin plots showing the distribution of Pearson correlation coefficients comparing the mean predicted per-residue fitness (calculated over a five-residue sliding window) from the βla-Aβ_42_-trained model with the ΔG^0^ contribution per position (also averaged over a five-residue sliding window) for Aβ_40_/Aβ_42_, hIAPP, α-synuclein, and TDP-43.

Given the correlation between the TPBLA fitness scores and C_crit_, we next employed a machine learning strategy to uncover the sequence rules underlying the variant fitness scores and, by extension, amyloid stability (*Materials and Methods*) (Fig. 5*A*). We developed two models. The first model was a random forest that was trained using embeddings derived from the ESM-2 (650 M) protein language model for each Aβ_42_ variant to predict βla-Aβ_42_ fitness score (5). The embeddings implicitly capture sequence features distinguishing variants, including longer-range (i + n) interactions through the attention layers in the transformer-based large language model (48). The second model, also a random forest, was trained using one-hot encoded amino acid identities, dipeptide composition, and additional sequence-derived features for each Aβ_42_ variant to predict βla-Aβ_42_ fitness score (49, 50). To avoid a prohibitive feature-to-observed-variable ratio and therefore risk of overfitting, we did not explicitly encode higher-order (i + n) pairings. Both models performed similarly when assessed using 10-fold cross-validation and the embeddings‐trained model achieved a mean R^2^ of 0.67 (SEM = 0.02), whereas the explicit feature model attained a mean R^2^ of 0.71 (SEM = 0.02) (*SI Appendix*, Fig. S13). Owing to its higher R^2^ and improved interpretability, we selected the explicit feature model for subsequent analyses, which we named ThermAL (Thermodynamics of Amyloid Landscapes) (Fig. 5). This model outperformed a panel of aggregation predictors, including AGGRESCAN (26), TANGO (24), Camsol (23), and Amylogram (25) at predicting the observed βla-Aβ_42_ profile (*SI Appendix*, Fig. S9). To interpret ThermAL, we employed SHapley Additive exPlanations (SHAP) analysis (51–53). This indicated that the strength of the model is determined by the polarity, β-sheet propensity, and side-chain bulkiness of each sequence (Fig. 5*B*). These features are consistent with those thought to drive amyloid stability (37). Analysis of the SHAP values revealed that high β‐sheet propensity and hydrophobicity are associated with lower variant fitness scores, whereas increased polarity correlates with higher fitness scores (*SI Appendix*, Fig. S14).

To evaluate the generalizability of ThermAL in identifying stabilizing and destabilizing regions of amyloid fibrils beyond Aβ_42_, we challenged the model to predict stabilizing regions in other amyloidogenic IDP sequences to which it had not been previously been exposed (α-synuclein, hIAPP, and TDP-43) (Fig. 5*C*). Using our pretrained model, we predicted variant fitness scores (and thus the effect of amino acid substitutions at each residue averaged over a five-residue window) for all single-point variant sequences to assess whether the physiochemical features driving amyloid stability are conserved across different intrinsically disordered amyloidogenic sequences (*Materials and Methods* and Fig. 5*C*) (37). We observed a good agreement between regions with high predicted changes in fitness score upon substitution and those regions that stabilize the fibrillar forms of each sequence determined using FoldX, suggesting that these features indeed represent the driving forces behind amyloid stability for these IDPs (*SI Appendix*, Figs. S15–S19). We posit that the poorer correlation for TDP-43 fibrils (*SI Appendix*, Fig. S19) may occur because the regions stabilizing its amyloid folds are more diverse within the family of TDP-43 fibril structures compared to the intrafamily diversity of Aβ_42_, hIAPP, and α-synuclein fibrils. Hence, we observe a good agreement between the model predictions for regions that stabilize hIAPP and α-synuclein amyloid fibrils (*SI Appendix*, Figs. S17 and S18, respectively), as well as for a class of TDP-43 amyloid structures which include PDBs 7PY2, 8QX9, and 7Q3U (54–56) (*SI Appendix*, Fig. S15).

## Discussion

We have previously shown that the TPBLA is a versatile assay able to report on a broad range of protein attributes including thermodynamic stability, self-interaction, small molecule binding, and amyloid formation (11–14). By embedding this assay as the fitness driver in a DMS format performed on solid medium, we show here that the assay can be used as a versatile and easy-to-perform screen, able to generate large-scale and high-quality datasets that capture both favorable and unfavorable attributes of variants of a POI. This platform not only facilitates detailed mechanistic insights into the sequence grammar governing the chosen attribute to be investigated but also allows the rapid generation of large, high-quality datasets using a well-characterized screen in the *E. coli* periplasm.

Our findings on Aβ_42_ demonstrate that, despite differences in expression systems (*E. coli* periplasm versus yeast cytoplasm) and construct designs [split β-lactamase versus N-terminal concatenation to DHFR (33)], the assays yield remarkably similar results, that highlight the two APRs in the protein sequence as important drivers of fitness. Our cross-validation analysis verifies that the assays are dominated by a single property, fibril stability, a finding that persists notwithstanding potential differences in variant expression. By contrast, Bolognesi et al., report that the Sup35N-Aβ_42_ dataset reports on the kinetics of fibril nucleation rather than thermodynamic stability (16). Hence, by combining the datasets, one based on fibril formation kinetics (the energy barrier to nucleation) and others based on fibril stability (the TPBLA or Aβ_42_-DHFR), the energy landscape of amyloid formation can be curated [a recent manuscript by Lehner et al. reports such an approach (57)]. Combined, our datasets reveal that APR1 plays a lesser role in nucleation compared to its influence on the thermodynamic stability of the endpoint fibril. By contrast, APR2 appears to play a strong role in both the kinetics of amyloid formation and in fibril stability. By identifying the scale to which β-sheet propensity, polarity, and hydrophobicity impact amyloid stability, our approach not only explains the early successes of aggregation prediction algorithms such as Camsol (23), TANGO (24), Aggrescan (26), and Amylogram (25), which were developed before high-resolution amyloid structures were available, but it also underscores the potential of the TPBLA for extending similar analyses to other amyloidogenic sequences. The high-throughput and resolution of the TPBLA approach also opens the door to more sophisticated formats, including deep indel mutagenesis [including variants such as the Osaka mutation E22Δ (58)] to further systematically dissect the thermodynamic grammar of amyloid formation or epistatic interactions through deeper mutagenesis (34). The periplasmic location of the assay would also permit proteins for which the presence of a disulfide bond is important for amyloidogencitiy (IAPP), or to include POI that are both disulfide bonded and structured in their native states [antibody light chains and β_2_-microglobulin (59–62)] which will further refine our understanding of the sequence dependence of amyloid formation and its stability.

The TPBLA platform leverages the intrinsic quality control mechanisms of *E. coli*, which selectively remove liabilities that compromise β‐lactamase function. Whether the selection pressure targets destabilizing substitutions in amyloid cores, reduces the self-association of antibody fragments that form less well-ordered aggregates, or probes for protein stability or solubility, we have demonstrated here [and hitherto (11–14)], the applicability of the TPBLA to capture different liabilities, demonstrating its utility in understanding and evolving a broad range of protein traits. Its versatility, not only for probing the aggregation and stability of amyloid-forming proteins but also to screen for chaperones, designed proteins, or small molecules able to bind to a POI [making use of protein coexpression in the bacterial periplasm or the permeability of the outer membrane to small molecules (<ca. 600 Da (63)] emphasize the value of the TPBLA in the protein discovery toolbox.

## Materials and Methods

Detailed explanations regarding deep mutational scan, Proteostat fluorescence, confocal microscopy, recombinant protein expression and purification, ThioT assay conditions, FoldX calculations, and Machine learning can be found in *SI Appendix*.

## Supplementary Material

Appendix 01 (PDF)

## Data Availability

The data associated with this paper are openly available from the University of Leeds Data Repository (https://doi.org/10.5518/1653) (64).

## References

[1] F. C. Bernstein , The protein data bank: A computer-based archival file for macromolecular structures. J. Mol. Biol. **112**, 535–542 (1977). 875032 10.1016/s0022-2836(77)80200-3

[2] J. Jumper , Highly accurate protein structure prediction with AlphaFold. Nature **596**, 583–589 (2021). 34265844 10.1038/s41586-021-03819-2 PMC8371605

[3] J. Abramson , Accurate structure prediction of biomolecular interactions with AlphaFold 3. Nature **630**, 493–500 (2024). 38718835 10.1038/s41586-024-07487-w PMC11168924

[4] B. E. Suzek, H. Huang, P. McGarvey, R. Mazumder, C. H. Wu, UniRef: Comprehensive and non-redundant UniProt reference clusters. Bioinformatics **23**, 1282–1288 (2007). 17379688 10.1093/bioinformatics/btm098

[5] T. Hayes , Simulating 500 million years of evolution with a language model. Science **387**, 850–858 (2025). 39818825 10.1126/science.ads0018

[6] M. Leander, Z. Liu, Q. Cui, S. Raman, Deep mutational scanning and machine learning reveal structural and molecular rules governing allosteric hotspots in homologous proteins. Elife **11**, e79932 (2022). 36226916 10.7554/eLife.79932 PMC9662819

[7] J. M. Schmiedel, B. Lehner, Determining protein structures using deep mutagenesis. Nat. Genet. **51**, 1177–1186 (2019). 31209395 10.1038/s41588-019-0431-x PMC7610650

[8] H. Wei, X. Li, Deep mutational scanning: A versatile tool in systematically mapping genotypes to phenotypes. Front. Genet. **14**, 1087267 (2023), 10.3389/fgene.2023.1087267. 36713072 PMC9878224

[9] S. D. Grosse, J. M. Gudgeon, Cost or price of sequencing? Implications for economic evaluations in genomic medicine. Genet. Med. **23**, 1833–1835 (2021). 34113006 10.1038/s41436-021-01223-9 PMC8487947

[10] A. J. Faure, B. Lehner, MoCHI: Neural networks to fit interpretable models and quantify energies, energetic couplings, epistasis, and allostery from deep mutational scanning data. Genome Biol. **25**, 303 (2024). 39617885 10.1186/s13059-024-03444-y PMC11610129

[11] J. S. Ebo , An in vivo platform to select and evolve aggregation-resistant proteins. Nat. Commun. **11**, 1816 (2020). 32286330 10.1038/s41467-020-15667-1 PMC7156504

[12] J. C. Saunders , An in vivo platform for identifying inhibitors of protein aggregation. Nat. Chem. Biol. **12**, 94–101 (2016). 26656088 10.1038/nchembio.1988 PMC4720988

[13] L. Foit , Optimizing protein stability in vivo. Mol. Cell **36**, 861–871 (2009). 20005848 10.1016/j.molcel.2009.11.022 PMC2818778

[14] N. Guthertz , The effect of mutation on an aggregation-prone protein: An in vivo, in vitro, and in silico analysis. Proc. Natl. Acad. Sci. U.S.A. **119**, e2200468119 (2022). 35613051 10.1073/pnas.2200468119 PMC9295795

[15] R. J. McLure, S. E. Radford, D. J. Brockwell, High-throughput directed evolution: A golden era for protein science. Trends Chem. **4**, 378–391 (2022).

[16] M. Seuma, A. J. Faure, M. Badia, B. Lehner, B. Bolognesi, The genetic landscape for amyloid beta fibril nucleation accurately discriminates familial Alzheimer’s disease mutations. Elife **10**, e63364 (2021). 33522485 10.7554/eLife.63364 PMC7943193

[17] A. M. Bendel , The genetic architecture of protein interaction affinity and specificity. Nat. Commun. **15**, 8868 (2024). 39402041 10.1038/s41467-024-53195-4 PMC11479274

[18] A. J. Faure , The genetic architecture of protein stability. Nature **634**, 995–1003 (2024). 39322666 10.1038/s41586-024-07966-0 PMC11499273

[19] L. Dewachter , Deep mutational scanning of essential bacterial proteins can guide antibiotic development. Nat. Commun. **14**, 241 (2023). 36646716 10.1038/s41467-023-35940-3 PMC9842644

[20] A. W. Golinski , High-throughput developability assays enable library-scale identification of producible protein scaffold variants. Proc. Natl. Acad. Sci. U.S.A. **118**, e2026658118 (2021). 34078670 10.1073/pnas.2026658118 PMC8201827

[21] A. D. Williams , Mapping Aβ amyloid fibril secondary structure using scanning proline mutagenesis. J. Mol. Biol. **335**, 833–842 (2004). 14687578 10.1016/j.jmb.2003.11.008

[22] L. O. Tjernberg , A molecular model of Alzheimer amyloid β-peptide fibril formation. J. Biol. Chem. **274**, 12619–12625 (1999). 10212241 10.1074/jbc.274.18.12619

[23] P. Sormanni, F. A. Aprile, M. Vendruscolo, The camsol method of rational design of protein mutants with enhanced solubility. J. Mol. Biol. **427**, 478–490 (2015). 25451785 10.1016/j.jmb.2014.09.026

[24] A. M. Fernández-Escamilla, F. Rousseau, J. Schymkowitz, L. Serrano, Prediction of sequence-dependent and mutational effects on the aggregation of peptides and proteins. Nat. Biotechnol. **22**, 1302–1306 (2004). 15361882 10.1038/nbt1012

[25] M. Burdukiewicz , Amyloidogenic motifs revealed by n-gram analysis. Sci. Rep. **7**, 12961 (2017). 29021608 10.1038/s41598-017-13210-9 PMC5636826

[26] O. Conchillo-Solé , AGGRESCAN: A server for the prediction and evaluation of ‘hot spots’ of aggregation in polypeptides. BMC Bioinformatics **8**, 65 (2007). 17324296 10.1186/1471-2105-8-65 PMC1828741

[27] A. Harmeier , Role of amyloid-β glycine 33 in oligomerization, toxicity, and neuronal plasticity. J. Neurosci. **29**, 7582–7590 (2009). 19515926 10.1523/JNEUROSCI.1336-09.2009 PMC6665404

[28] B. Houben , Autonomous aggregation suppression by acidic residues explains why chaperones favour basic residues. EMBO J. **39**, e102864 (2020). 32237079 10.15252/embj.2019102864 PMC7265246

[29] S. Lifson, C. Sander, Antiparallel and parallel β-strands differ in amino acid residue preferences. Nature **282**, 109–111 (1979). 503185 10.1038/282109a0

[30] H. Hampel , The amyloid-β pathway in Alzheimer’s disease. Mol. Psychiatry **26**, 5481–5503 (2021). 34456336 10.1038/s41380-021-01249-0 PMC8758495

[31] S. Navarro, S. Ventura, Fluorescent dye ProteoStat to detect and discriminate intracellular amyloid-like aggregates in Escherichia coli. Biotechnol. J. **9**, 1259–1266 (2014). 25112199 10.1002/biot.201400291

[32] S. Linse, “Expression and purification of intrinsically disordered Aβ peptide and setup of reproducible aggregation kinetics experiment” in Intrinsically Disordered Proteins: Methods and Protocols, B. B. Kragelund, K. Skriver, Eds. (Springer, 2020), pp. 731–754. 10.1007/978-1-0716-0524-0_38 32696387

[33] V. E. Gray , Elucidating the molecular determinants of Aβ aggregation with deep mutational scanning. G3 (Bethesda) **9**, 3683–3689 (2019). 31558564 10.1534/g3.119.400535 PMC6829127

[34] M. Seuma, B. Lehner, B. Bolognesi, An atlas of amyloid aggregation: The impact of substitutions, insertions, deletions and truncations on amyloid beta fibril nucleation. Nat. Commun. **13**, 7084 (2022). 36400770 10.1038/s41467-022-34742-3 PMC9674652

[35] M. R. Sawaya, M. P. Hughes, J. A. Rodriguez, R. Riek, D. S. Eisenberg, The expanding amyloid family: Structure, stability, function, and pathogenesis. Cell **184**, 4857–4873 (2021). 34534463 10.1016/j.cell.2021.08.013 PMC8772536

[36] J. Schymkowitz , The foldX web server: An online force field. Nucleic Acids Res. **33** (suppl. 2), W382–W388 (2005). 15980494 10.1093/nar/gki387 PMC1160148

[37] R. Van der Kant, N. Louros, J. Schymkowitz, F. Rousseau, Thermodynamic analysis of amyloid fibril structures reveals a common framework for stability in amyloid polymorphs. Structure **30**, 1178–1189.e3 (2022). 35609599 10.1016/j.str.2022.05.002

[38] J. Connor, Structural and thermodynamic classification of amyloid polymorphs. Structure, 10.1016/j.str.2025.07.005 (2025). 40730163

[39] A. Fernandez , Cryo-EM structures of amyloid-β and tau filaments in Down syndrome. Nat. Struct. Mol. Biol. **31**, 903–909 (2024). 38553642 10.1038/s41594-024-01252-3 PMC11189299

[40] L. Gremer , Fibril structure of amyloid-β(1–42) by cryo-electron microscopy. Science **358**, 116–119 (2017). 28882996 10.1126/science.aao2825 PMC6080689

[41] M. R. Elkins , Structural polymorphism of Alzheimer’s β-amyloid fibrils as controlled by an E22 switch: A solid-state NMR study. J. Am. Chem. Soc. **138**, 9840–9852 (2016). 27414264 10.1021/jacs.6b03715 PMC5149419

[42] M. Zielinski , Cryo-EM of Aβ fibrils from mouse models find tg-APPArcSwe fibrils resemble those found in patients with sporadic Alzheimer’s disease. Nat. Neurosci. **26**, 2073–2080 (2023). 37973869 10.1038/s41593-023-01484-4 PMC10689242

[43] R. K. Norrild, N. Vettore, A. Coden, W. F. Xue, A. K. Buell, Thermodynamics of amyloid fibril formation from non-equilibrium experiments of growth and dissociation. Biophys. Chem. **271**, 106549 (2021). 33578107 10.1016/j.bpc.2021.106549

[44] M. Novo, S. Freire, W. Al-Soufi, Critical aggregation concentration for the formation of early amyloid-β (1–42) oligomers. Sci. Rep. **8**, 1783 (2018). 29379133 10.1038/s41598-018-19961-3 PMC5789034

[45] S. Illodo, W. Al-Soufi, M. Novo, Critical aggregation concentration and reversibility of amyloid-β (1–40) oligomers. Arch. Biochem. Biophys. **761**, 110179 (2024). 39393664 10.1016/j.abb.2024.110179

[46] C. Xue, T. Y. Lin, D. Chang, Z. Guo, Thioflavin T as an amyloid dye: Fibril quantification, optimal concentration and effect on aggregation. R. Soc. Open Sci. **4**, 160696 (2017). 28280572 10.1098/rsos.160696 PMC5319338

[47] A. J. Dear , The catalytic nature of protein aggregation. J. Chem. Phys. **152**, 045101 (2020). 32007046 10.1063/1.5133635 PMC7377910

[48] A. Vaswani , Attention is all you need. arXiv [Preprint] (2017). 10.48550/arXiv.1706.03762 (Accessed 3 September 2025).

[49] J. M. Walker, Ed., The Proteomics Protocols Handbook (Humana Press, 2005).

[50] Z. Chen , iFeature: A Python package and web server for feature extraction and selection from protein and peptide sequences. Bioinformatics **34**, 2499–2502 (2018). 29528364 10.1093/bioinformatics/bty140 PMC6658705

[51] A. V. Ponce-Bobadilla, V. Schmitt, C. S. Maier, S. Mensing, S. Stodtmann, Practical guide to SHAP analysis: Explaining supervised machine learning model predictions in drug development. Clin. Transl. Sci. **17**, e70056 (2024). 39463176 10.1111/cts.70056 PMC11513550

[52] R. Harun, J. Lu, N. Kassir, W. Zhang, Machine learning–based quantification of patient factors impacting remission in patients with ulcerative colitis: Insights from etrolizumab phase III clinical trials. Clin. Pharmacol. Ther. **115**, 815–824 (2024). 37828747 10.1002/cpt.3076

[53] S. Basu , Predicting disease activity in patients with multiple sclerosis: An explainable machine-learning approach in the Mavenclad trials. CPT Pharmacometrics Syst. Pharmacol. **11**, 843–853 (2022). 35521742 10.1002/psp4.12796 PMC9286719

[54] K. Sharma , Cryo-EM observation of the amyloid key structure of polymorphic TDP-43 amyloid fibrils. Nat. Commun. **15**, 486 (2024). 38212334 10.1038/s41467-023-44489-0 PMC10784485

[55] S. T. Kumar , Seeding the aggregation of TDP-43 requires post-fibrillization proteolytic cleavage. Nat. Neurosci. **26**, 983–986 (2023). 37248338 10.1038/s41593-023-01341-4 PMC10244175

[56] D. Arseni , Structure of pathological TDP-43 filaments from ALS with FTLD. Nature **601**, 139–143 (2022). 34880495 10.1038/s41586-021-04199-3 PMC7612255

[57] A. Arutyunyan, M. Seuma, A. J. Faure, B. Bolognesi, B. Lehner, Massively parallel genetic perturbation suggests the energetic structure of an amyloid-β transition state. Sci. Adv. **11**, eadv1422 (2025). 40498820 10.1126/sciadv.adv1422 PMC12153979

[58] T. Tomiyama , A new amyloid β variant favoring oligomerization in Alzheimer’s-type dementia. Ann. Neurol. **63**, 377–387 (2008). 18300294 10.1002/ana.21321

[59] A. I. P. Taylor , Kinetic steering of amyloid formation and polymorphism by canagliflozin, a type-2 diabetes drug. J. Am. Chem. Soc. **147**, 11859–11878 (2025). 39985130 10.1021/jacs.4c16743 PMC11987024

[60] Y. Xu , Tuning the rate of aggregation of hIAPP into amyloid using small-molecule modulators of assembly. Nat. Commun. **13**, 1040 (2022). 35210421 10.1038/s41467-022-28660-7 PMC8873464

[61] G. Merlini , Systemic immunoglobulin light chain amyloidosis. Nat. Rev. Dis. Primers **4**, 1–19 (2018). 30361521 10.1038/s41572-018-0034-3

[62] M. G. Iadanza , The structure of a β2-microglobulin fibril suggests a molecular basis for its amyloid polymorphism. Nat. Commun. **9**, 4517 (2018). 30375379 10.1038/s41467-018-06761-6 PMC6207761

[63] H. Nikaido, Molecular basis of bacterial outer membrane permeability revisited. Microbiol. Mol. Biol. Rev. **67**, 593–656 (2003). 14665678 10.1128/MMBR.67.4.593-656.2003 PMC309051

[64] C. McKay , Data from “Employing deep mutational scanning in the Escherichia coli periplasm to decode the thermodynamic landscape for amyloid formation.” Research Data Leeds Repository. 10.5518/1653. Deposited 26 June 2025. PMC12478104 40961135

