# Supplementary material for: Employing deep mutational scanning in the Escherichia coli periplasm to decode the thermodynamic landscape for amyloid formation
Source: Proc Natl Acad Sci U S A. 2025 Sep 17;122(38):e2516165122. doi: 10.1073/pnas.2516165122 (PMC12478104; doi:10.1073/pnas.2516165122)
Supplement: Supplementary file 1 — Appendix 01 (PDF) [file pnas.2516165122.sapp.pdf]

Supplementary Methods and Figures

Employing deep mutational scanning in the *Escherichia coli* periplasm to decode the thermodynamic landscape for amyloid formation

Author Information

Conor E. McKay[1,2], Miles Deans[1,2], Jack Connor[1,2] Janet C. Saunders [3,4], Christopher Lloyd[3], Sheena E. Radford[1,2*] and David J. Brockwell[1,2*].

[1] Astbury Centre for Structural and Molecular Biology, University of Leeds;[2] School of Molecular and Cellular Biology, Faculty of Biological Sciences, University of Leeds, Leeds, LS2 9JT, UK, [3]The Discovery Centre, AstraZeneca, Cambridge CB2 0AA, U.K.;[4]Present address: Immunocore, 92 Park Dr, Milton, Abingdon OX14 4RY

*Corresponding authors: s.e.radford@leeds.ac.uk d.j.brockwell@leeds.ac.uk

This PDF file includes:

Supporting text, Methods

Figures S1 to S19

Table S1

Supplementary References

Supplementary Methods

Construction of β-lactamase $A\beta_{42}$ Library

A saturation library comprising every single amino-acid substitution at each residue of $A\beta_{42}$, flanked by BsaI sites was purchased from TWIST Bioscience. The library was cloned into a previously described β-lactamase expression pBR322 vector modified to allow in-frame insertion into the 28 residue Gly-Ser linker region via BsaI sites by Golden Gate assembly using the NEB golden gate assembly kit. The library was then purified using a Qiagen PCR purification kit and eluted in water. The library was electroporated into Agilent-provided electrocompetent TG1 cells using a MicroPulser Electroporator (BioRad) (2.5 kV field strength, 335 Ω resistance and 15 µF capacitance) in a 1.0mm cuvette (BioRad) and the cells were then incubated for 1 h, at 37 °C shaking at 250 rpm in 950 µL Agilent-provided recovery medium (20g/L tryptone, 5g/L yeast extract, 8.5mM NaCl). Cells were then plated on agar plates containing 10 µg mL^{-1} tetracycline. After incubation overnight, plasmid DNA was extracted using a Zymopure Midi Prep Kit.

Deep Substitutional Scan of $A\beta_{42}$ Using the TPBLA

50 µL of supercompetent *E. coli* (SCS1) were incubated with 200 ng of purified βla-$A\beta_{42}$ library on ice for 30 min before heat shocking at 42 °C for 45 sec. After 5 min incubation on ice, 950 µL SOC Agilent-provided recovery medium (20g/L tryptone, 5g/L yeast extract, 8.5mM NaCl) was added and bacteria were incubated (37 °C, 200 rpm) for 1 hour. 9.05 mL SOC medium containing 10 µg mL^{-1} tetracycline was then added to the culture. Cells were incubated until OD_{600} = 0.1 and β-lactamase expression was induced with 0.075 % (w/v) arabinose. After 1 h, bacteria were plated onto bioassay plates containing 2.5 % (w/v) Luria Broth, 1.5 % (w/v) agar, 10 µg mL^{-1} tetracycline, 0.075 % (w/v) arabinose and either 0, 5, 10, 15, 20, 40, 60, 80, or 100 µg mL^{-1} ampicillin.

Preparation of Samples of Next Generation Sequencing

DNA from each ampicillin selection plate was extracted using a Zymopure Midi Prep Kit. Illumina partial adapters were added to each sample by PCR using custom primers binding the G/S linkers flanking $A\beta_{42}$. The expected PCR product of 217 base pairs was confirmed using agarose gel electrophoresis. DNA was excised and purified using a NEB gel extraction kit and quantified using a Qubit fluorometer (Thermo Fisher Scientific). Each amplicon was sent for Azenta ampliconEZ next generation sequencing at a concentration of 20 ng μL^{-1}.

Next Generation Sequencing Processing and Error Thresholding

Paired end reads for each sequencing reaction, which also corresponds to a single ampicillin concentration used for selection, were obtained in FastQ format. Reads were merged using BBMerge (1) and quality filtered using Cutadapt (2), with merged reads requiring a Phred quality score of greater than 40 to be considered. Quality thresholded merged reads were then aligned to the wild-type $A\beta_{42}$ gene sequence using Bowtie2 (3), with those which were not exactly 126 bp in length being discarded and the number of reads for each variant was computed. The impact of sequencing errors was evaluated by measuring the number of amino acid variants detected in part of the invariant G/S linker sequence C-terminal to $A\beta_{42}$, which was included in the sequencing amplicon, reflecting the error rate in sequencing. To be considered above the threshold for each sequencing reaction, a variant must exhibit a number of reads greater than the mean number of reads acquired by variants at positions G43 and S44 (which are parts of the invariant linker purposefully included in the amplicon for thresholding) plus two times the standard deviation of reads acquired by variants of positions G43 and S44.

Assigning Variant Fitness Score

To derive the variant fitness score for each variant, the following equations were used:

$$Enrichment\ Score_{x\mu g/mL} = \frac{((Number\ of\ variant\ reads\ \div Total\ number\ of\ reads) \times\ Number\ of\ variants)_{x\mu g/mL}}{(Number\ of\ variant\ reads\ \div Total\ number\ of\ reads)\ \times\ Number\ of\ variants)_{0\mu g/mL}}$$

$$Variant\ Fitness = log_2(\frac{\left(\sum_{i=1}^{9} \frac{y_i + y_{i+1}}{2}\ (x_{i+1} - x_i)\right)_{variant}}{\left(\sum_{i=1}^{9} \frac{y_i + y_{i+1}}{2}\ (x_{i+1} - x_i)\right)_{Wild-Type}})$$

The top equation gives an enrichment score for each variant as a function of ampicillin concentration. This involves dividing the number of reads for the variant of interest by the total number of reads in that sequencing reaction and multiplying by the total number of variants present in that sequencing file. This value is then divided by the same value in the control naïve sequencing reaction for that biological replicate to give a per [ampicillin] enrichment score. These scores are then used to create a trapezium with [ampicillin] on the horizontal axis and enrichment score on the vertical axis which is integrated using the trapezoidal rule where x_i and y_i are the ampicillin concentration and enrichment score for that concentration, respectively. The area under the curve (AUC) for each variant is then divided by the AUC for wild-type and taking Log_2 of this value yields variant fitness score. This analysis was conducted for each biological repeat (n=3) and averaged to obtain a final variant fitness score. Variants were required to be in two of three biological repeats at 0 µg mL^{-1} ampicillin to be considered. Those that only appeared on one were excluded from consideration. An example of this processing is provided in Supplementary Figure 3.

To minimise sequencing errors being assigned as true variants, the standard error of all variants between replicates was calculated and the mean ($mean_{SEM}$) and standard deviation (SD_{SEM}) of the population of standard errors were calculated.

Variants with SEM > $mean_{SEM}$ + 2 SD_{SEM} were removed from consideration. This led to variants D23P, H6P, E22V, A21E, Q15C and D23L being removed from the dataset.

In Cell Proteostat Fluoresence

E. coli SCS1 cells transformed with βla-A$β_{42}$, βla-G/S, or bacteria expressing mA$β_{42}$ aggrgates in the cytoplasm (peTSAC-mA$β_{42}$, kindly provided by Sara Linse (Lund University)) were incubated for 16 h overnight (37 ˚C) following the same DMS protocol described above. Bacteria were then harvested from plates by scraping and washed, and diluted to an OD_{600} of 0.4 in phosphate buffer saline (Fisher Scientific: 8g/L sodium chloride, 0.2g/L potassium chloride, 1.15g/L di-sodium hydrogen phosphate, 0.2g/L potassium dihydrogen phosphate). Proteostat dye (Enzo Life Sciences) was added to samples at 1:5000 dilution and incubated for 30 min at room temperature. Fluorescence emission of samples was measured at 570 nm upon excitation at 488 nm using a ClarioStar microplate reader. Shapiro–Wilk normality tests were performed on all four datasets. In each case, the test failed to reject the null hypothesis of normality, indicating no statistically significant departure from a Gaussian distribution. Consequently, Statistical significance was determined using an unpaired t-test between βla-G/S and βla-A$β_{42}$, and a paired t-test between uninduced βla-A$β_{42}$ and βla-A$β_{42}$.

Confocal images of the bacteria prepared as described above were also captured using a Zeiss LSM880 inverted confocal microscope with a 63x objective. Slides were prepared by coating with 0.1% (w/v) poly-L-lysine and air-dried for 30 min before applying 20 µL of the cell sample. Confocal fluorescence images were acquired using 488 nm excitation and a 500–600 nm filter for fluorescence detection.

Calulation of Per Residue Amyloid Stability Using FoldX

FoldX was used to calculate the contribution of each residue to the total free energy (ΔG°) for different amyloid structures. To account for published structures differing in the number of cross-β layers, each structure was extended to a layer depth of 10 so that each fibril comprised a stack of 10 monomers. Only the internal 8 layers were used for FoldX calculations. Most published structures do not include water molecules, so all water atoms were removed prior to thermodynamic analysis. A Python script using the pyFoldX package (4) was used to automate FoldX calculations of ΔG° enabling rapid analysis. First, the command RepairPDB was used to minimise the energy of the structures. Next, the script runs the command, SequenceDetail, which returns the FoldX calculated energy terms averaged for each residue. SequenceDetail calculates the total ΔG° contribution for each residue as follows:

$$\Delta G^\circ = \Delta G^\circ_{vdw} + \Delta G^\circ_{solvH} + \Delta G^\circ_{solvP} + \Delta G^\circ_{wb} + \Delta G^\circ_{hbond} + \Delta G^\circ_{el} + \Delta G^\circ_{kon} + \Delta G^\circ_{clash} + \Delta S^\circ_{mc} + \Delta S^\circ_{sc}$$

The total contribution to free energy for each residue was calculated by summing the individual energy terms; the sum of Van der Waals contributions ($\Delta G°_{vdw}$), the solvation energy for polar ($\Delta G°_{solvH}$) and polar groups ($\Delta G°_{solvP}$), water bridges ($\Delta G°_{wb}$), hydrogen bonding ($\Delta G°_{hbond}$), electrostatic interactions ($\Delta G°_{el}$), a second metric measuring the electrostatic interactions between different polypeptide chains ($\Delta G°_{kon}$), steric clashes ($\Delta G°_{clash}$), and the entropy of the main ($\Delta S°_{mc}$) and side chains ($\Delta S°_{sc}$). The total contribution to free energy does not include the backbone Van der Waals clashes.

Site-Directed Mutagenesis of NT*$_{FlSP}$-Aβ_{42}

A pT7 plasmid containing the gene for Aβ_{42} fused via a TEV protease recognition site to NT*FlSp (a pH-insensitive, solubility-enhancing variant of the N-terminal domain of flagelliform spider silk protein) was kindly donated by Dr. Henrik Biverstål (Karolinska Institutet). The TEV protease recognition site in this construct contains a non-canonical aspartate residue at the P1' position, which slightly reduces cleavage efficiency compared to the canonical site, but is still able to cleave the fusion protein to release native Aβ_{42} for downstream applications (5) (6) (7) (8). The 6xHis tag facilitates affinity purification of the expressed fusion protein using immobilized metal affinity chromatography (IMAC). This modular design enables expression and purification of the Aβ_{42} peptide, with the option to remove the NT*FlSp solubility tag by TEV protease treatment to yield native Aβ_{42} wihout an initiating methionine for downstream experiments.

Expression and Purification Aβ_{42} Monomer

A single colony of *E. coli* BL21 (DE3) freshly transformed with a suitable pT7 NT*$_{FlSP}$-Aβ_{42} plasmid was used to inoculate an overnight culture in 2.5% (w/v) LB. This culture was diluted 1:1000 into auto-induction medium (50 µg mL^{-1} kanamycin) and incubated for 72 h (16°C, 200 rpm). Cells were harvested by centrifugation at 4000 *g* at 4 °C and the pellet was frozen. Pellets were incubated with 25 mM Tris, 2 mM $MgCl_2$, pH 8. DNase (20 µg mL^{-1}) and lysozyme (100 µg mL^{-1}) were added and bacteria incubated under roller agitation for 1 hour. Cultures were then homogenised and passed through a cell disruptor (30 kpsi, 25 °C), urea added to a final concentration of 6 M and the sample stirred (30 min, 25 °C). After clarification by centrifugation (24,000g, 25 °C, 30 min) the lysate was filtered and loaded onto a 5 mL Ni-NTA column pre-equilibrated with 25 mM Tris, 300 mM NaCl, 10 mM imidazole, 8 M urea, pH 8. The sample was eluted with a single step using 25 mM Tris, 300 mM NaCl, 250 mM imidazole, 8 M urea, pH 8. For TEV cleavage, the sample was diluted to reduce the urea concentration from 8 M to 1 M. Reagents were then added to achieve a final concentration of 1 mM DTT, 0.5 mM EDTA, and 1 M urea, pH 8. TEV protease (expressed and purfied according to reference: (6)) was added at a 20:1 (w/w) (NT*$_{FlSP}$-Aβ_{42}:TEV). Cleavage was performed under roller agitation at 4 °C for 18 h 55). Guanidine hydrochloride (GuHCl) was added to samples (final concentration 6 M GuHCl) and Aβ_{42} was separated from TEV and NT*$_{FlSP}$ on a Superdex75 26/600PG size exclusion column, pre-equilibrated with 20 mM Tris, 300 mM NaCl, 6M GuHCl, pH 8. Fractions containing Aβ_{42} (identified by SDS-PAGE), were pooled, dialysed into 5 mM ammonium bicarbonate using Spectrum Labs 1 kDa dialysis tubing and lyophilised.

Aggregation Kinetics and Determination of C_{Crit}

Pure monomeric $A\beta_{42}$ was obtained by dissolving freeze-dried protein stock in 6 M GuHCl at 4 °C for 20 min under agitation, to dissociate any pre-existing aggregates. The sample was then passed through a 0.22 µm filter to remove insoluble aggregates. The filtered solution was injected onto a Superdex 75 10/300 size exclusion column pre-equilibrated with 20 mM NaH_2PO_4, 0.2 mM EDTA, 0.02 % NaN_3 (w/v), pH 8.0. The concentration of the eluted $A\beta_{42}$ monomer was determined using UV-spectrophotometry using molar extinction co-efficient of 1490 $M^{-1}cm^{-1}$ for wild-type $A\beta_{42}$ and all variants except V24Y (ε_{280} =2980$M^{-1}cm^{-1}$).

Aggregation of $A\beta_{42}$ was monitored using ThioT fluorescence. Purified $A\beta_{42}$ was diluted on ice in 20 mM NaH_2PO_4, 0.2 mM EDTA, 0.02 % (w/v) NaN_3, pH 8.0, to concentrations spanning 0.5-24µM, with a constant concentration of 10 µM ThioT. Plates (Corning 3881: 96 well, half-area, non-binding polystyrene) containing $A\beta_{42}$ sample were incubated quiescently, at 37 °C. ThioT fluorescence (excitation at 440 nm, emission at 480 nm) was measured using a FLUOstar Omega platereader, with the signal normalized against a blank containing 20 mM NaH_2PO_4, 0.2 mM EDTA, 0.02 % (w/v) NaN_3, 10 µM ThioT, pH 8.0.

Extrapolating C_{crit} Using the Protein Concentration Dependence of ThioT Fluoresence

Endpoint ThioT fluorescence intensity was determined for samples for which the ThioT fluorescence had reached a clear plateau by the end of the experiment. This value was obtained for at least two technical replicates per experiment and two biological repeats, and used to compute the mean fluorescence intensity and standard error of the mean (SEM) for each $A\beta_{42}$ concentration. Weighted linear regression analysis was used to extrapolate the data to determine the x-axis intercept which is representative of the critical concentration (C_{crit}) for amyloid formation for each variant:

$$w_i = \frac{1}{{\sigma_i}^2} \quad \text{and} \quad SSR_{weighted} = \sum w_i(y_i - (mx_i + c))^2$$

where w_i is the weight at concentration at datapoint i. y_i is the observed ThioT fluorescence intensity, x_i is the $A\beta_{42}$ concentration (µM), m and c are the slope and intercept, respectively. $SSR_{weighted}$ is the weighted sum of squared residuals which is minimised as part of the fitting process.

The error in the fit was also accounted for using the curve_fit package in SciPy (9). Briefly, the error in C_{crit} is determined by propagating the uncertainties in m and c. During the fitting process, the curve_fit function returns both the best-fit parameters and a covariance matrix (pcov), which contains the variances for m and c on the diagonal. To obtain the error in C_{crit} standard error propagation was applied:

$$\sigma_{C_{crit}} = \sqrt{(\frac{\sigma_c}{m})^2 + (\frac{c.\sigma_m}{m^2})^2}$$

The two biological repeats are then combined, with SEM considered:

$$w_1 = \frac{1}{\sigma^2{}_{R1}}$$

$$w_2 = \frac{1}{\sigma^2{}_{R2}}$$

$$C_{Crit\ (combined)} = \frac{w_1.C_{critR1} + w_2.C_{critR2}}{w_1 + w_2}$$

$$\sigma_{combined} = \sqrt{\frac{1}{w_1 + w_2}}$$

Extracting κ and λ from Aβ$_{42}$ Aggregation Kinetics

ThioT fluorescence intensity versus time using at 8 µM initial Aβ$_{42}$ monomer were normalised to the maximum ThioT fluorescence intensity at the end of the experiment. To extract κ and λ the following equation was fitted to the data, using least squares fitting to optimise the fit:

$$y = 1 - (1 + \frac{\lambda^2}{1.2\kappa^2}(e^{\kappa t} + e^{-\kappa t} - 2))^{-\theta}$$

where θ was assumed to be 0.6 as Aβ$_{42}$ kinetics obey secondary nucleation governed aggregation (10).

Calculation of Physiochemical Sequence Feature

Prior to machine learning, a set of sequence descriptors were attributed to each Aβ$_{42}$ single residue substitution. Amino acid composition, dipeptide composition and AAindex features were obtained using iFeature (11). The latter was later removed from training due to overfitting to the numerous present features. The AAindex features were replaced with 7 simple descriptors: bulkiness, β-turn propensity, coil propensity, β-sheet propensity, α-helix propensity, hydrophobicity, and polarity (12). These descriptors were derived from published amino acid property atlases available on Expasy(13) and computed for each residue using a 9-residue sliding window. The feature value at each sequence position was determined by calculating a sliding average across the window. To obtain a single sequence-level representation, the area under the curve (AUC) of each feature's per-position curve was computed, yielding one numerical value per sequence for each descriptor. All features were scaled using StandardScaler present within the Scikit Learn python package, centring the mean of the features to 0 and ensuring a standard deviation of 1(14). This transformation was applied to prevent features with larger magnitudes from disproportionately influencing the machine learning model.

A random forest model was trained using the features outlined above to predict the target variable: variant fitness using scikit-learn (14). The model was assessed by 10-fold cross validation and the median performance model (R^2=0.72, SEM=0.0183) was carried forward.

To discern the features driving the power of the model, and therefore amyloid stability, SHAP analysis was employed: a game theoretic framework, which has previously been used to derive interpretability behind machine learning models (15). A comprehensive overview of SHAP analysis has been outlined previously (16). Briefly, the SHAP values for all features j of variant x, $\Phi_{i,j}$ satisfy the following formula:

$$f(x) = \Phi_0 + \sum_{Features} \Phi_{x,j}$$

where f(x) is the model prediction for variant x variant fitness score, Φ_0 is the average prediction for variant fitness score and $\sum_{Features} \Phi_{x,j}$ is the sum of all SHAP values for variant x. This SHAP value for a given variant x describes the extent each feature contributed to the cause difference between the average predicted variant fitness score and variant fitness score for variant x.

To test the model's ability to identify stabilising and destabilising regions in sequences other than $A\beta_{42}$, sequence features for α-synuclein, hiAPP and TDP-43 were derived and scaled as described above. The pre-trained $A\beta_{42}$ model was then used to compute the change in predicted variant fitness from the wild-type test sequence for each single point substitution based on the sequence descriptors for each single point substitution of the sequence. The average per residue fitness score was then computed for each position and correlated against the FoldX calculated energy terms averaged for each residue for each PDB available of each sequence ($A\beta_{42}$=26, hIAPP=15, α-synuclein=90, TDP-43=11).

Sequence features for hIAPP, α-synuclein and TDP-43 were derived and scaled sequence as for $A\beta_{42}$ as above. Using the pre-trained $A\beta_{42}$ model, we computed the change in predicted variant fitness for each single-point substitution relative to the wild type, based on the corresponding sequence descriptors. We then averaged these fitness scores per residue and correlated them with FoldX-calculated energy terms, averaged across the available PDB structures ($A\beta_{42}$ = 26, hIAPP = 15, α-synuclein = 90, TDP-43 = 11).

Supplementary Table

Supplementary Table 1: Aβ$_{42}$ variants associated with Alzheimer's disease curated from the Alzforum database, alongside their experimentally determined βla-Aβ$_{42}$ variant fitness scores.

* A2T Is associated with protection from Alzheimer's disease.

Mutation	Observed TPBLA Variant Fitness
**A2V**	-1.00
**A2T***	-0.74
**H6R**	-1.26
**D7N**	-0.58
**D7H**	-1.48
**E11K (Leuven)**	-0.05
**E11V**	-1.64
**K16Q**	-1.21
**K16N**	0.17
**L17V (Greek)**	-0.86
**ΔF19-V24 (Uppsala)**	Not in the dataset
**A21G (Flemish)**	1.16
**E22Q (Dutch)**	-1.69
**E22G (Arctic)**	-1.10
**E22K (Itailian)**	-0.80
**E22Δ (Osaka)**	Not in the dataset
**E22N**	0.41
**D23N (Iowa)**	-0.98
**N27D**	-1.03
**L34V (Piedmont)**	-0.47
**G38S**	-1.05
**A42T**	-1.06

Supplementary Figures

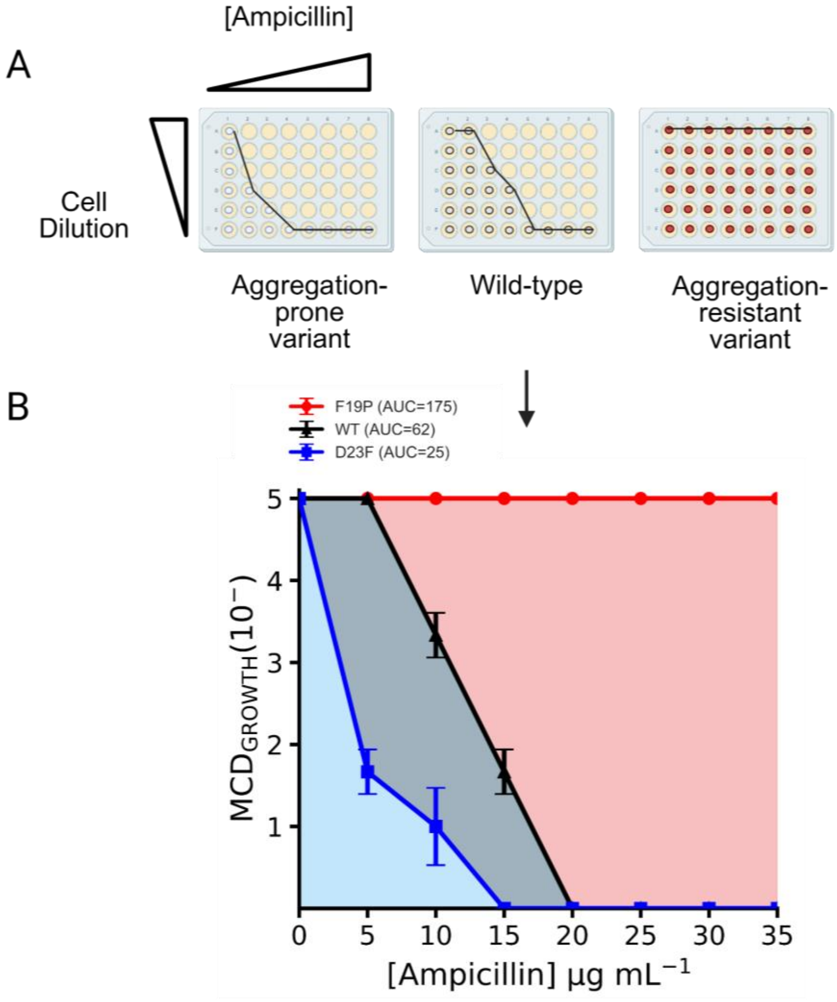

Supplementary Figure 1. TPBLA in the 48-well format: **(A)** *E. coli* expressing the protein of interest (POI) are plated onto a 48-well plate containing increasing ampicillin concentrations and increasing serial cell dilutions (10^0–10^5). For each POI variant, the highest dilution showing growth at each antibiotic concentration after overnight incubation (Methods) is plotted to yield a survival curve (black line). **(B)** The area under that curve (AUC) yields a quantitative read-out of the effect of the sequence of the POI on β-lactamase activity. Representative survival curves for an aggregation-prone variant (D23F, blue; AUC = 25), wild-type (grey; AUC = 62), and an aggregation-resistant variant (F19P, red; AUC = 175) of Aβ_{42}. Smaller AUC values correspond to greater aggregation because of loss of β-lactamase function.

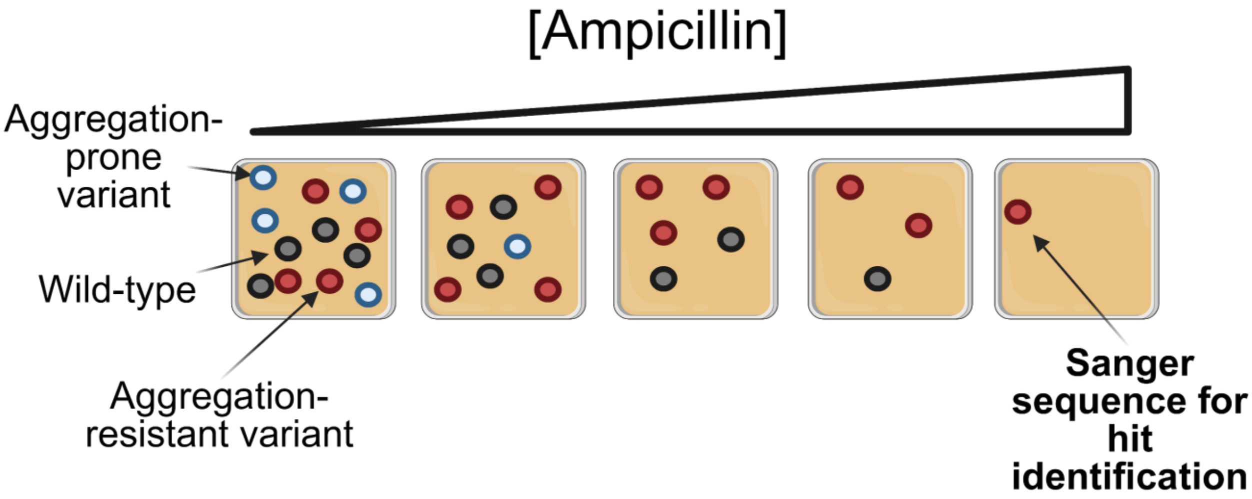

Supplementary Figure 2. Application of the second-generation of the TPBLA as a directed evolution screen. A library of variants is plated on bioassay plates each with an increased ampicillin concentration. Sanger sequencing of colonies present on the highest [ampicillin] allows for identification of variants with favorable properties.

1) *Read Processing and Enrichment Calculation*

[Ampicillin] µg/mL	F19P Read Counts	Number of Variants	Number of Sequences	Per-Plate Score	Enrichment Score
0	41	760	61626	0.51	1
5	115	715	49686	1.65	3.24
10	26	719	49547	0.38	0.75
15	165	708	56118	2.08	4.08
20	211	671	47459	2.98	5.84
40	451	421	37274	5.09	9.98
60	1437	510	44334	16.53	32.41
80	797	365	30750	9.46	18.55
100	938	518	29509	16.47	32.29

2) *Integration of Enrichment Throughout Selection*

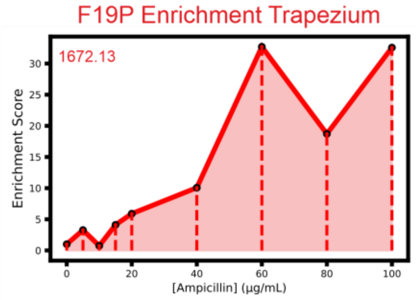

3) *Variant Fitness Score: Enrichment Relative To Wild-Type*

F19P Integrated Enrichment Trapezium

F19P Variant Fitness = $\mathrm{Log}_2(1672.13/45.96)$

F19P Variant Fitness = 5.19

WT Integrated Enrichment Trapezium

4) *Averaging Between Biological Repeats*

F19P Variant Fitness = (5.19 + 3.52 + 4.03)/3 = 4.25 +- 0.49

R1 R2 R3

Supplementary Figure 3: Schematic showing how variant fitness score is obtained in the TPBLA in DMS format, using F19P as an example. Raw FASTQ reads from the tripartite β-lactamase assay (TPBLA) are first sorted into counts per $A\beta_{42}$ variant on each ampicillin concentration plate (0, 10, 20, 40, 60, 80, 100 µg/mL ampicillin). As described in Methods, for each plate a "per-plate score" is computed by dividing each variant's read count by the total reads on that plate and then multiplying by the total number of variants present. The 0 µg/mL plate serves as the naïve (no-Amp) control; each variant's per-plate score at higher [Amp] is divided by its per-plate score on the 0 µg/mL plate to yield an enrichment value at that concentration. Enrichment is plotted versus [Amp], and the area under this curve is calculated by the trapezoidal rule to give a single integrated enrichment value per variant. The respective integrated enrichment value for wild-type $A\beta_{42}$ is determined and the trapezium for each variant is divided by the wild-type. Finally, each variant's $\log_2$-fitness is averaged over three independent biological replicates (n = 3), and the mean ± SD is reported.

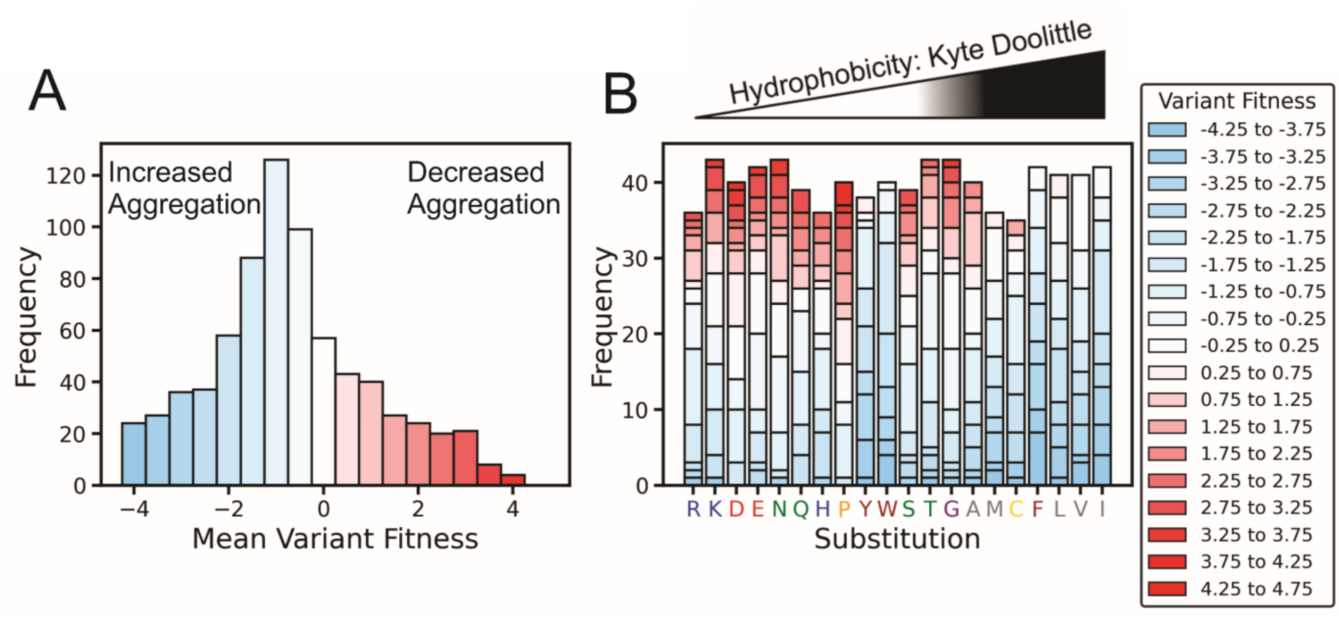

Supplementary Figure 4: Selection of Aβ_{42} variants using the TBPLA in DMS format. (A) Frequency distribution of variant fitness scores. Negative values indicate increased aggregation and positive values indicate decreased aggregation. **(B)** Effect of substitution to each amino acid on variant fitness scores, with amino acids ordered by Kyte Doolittle hydrophobicity. The scaling for variant fitness score is depicted in the adjacent colour map legend.

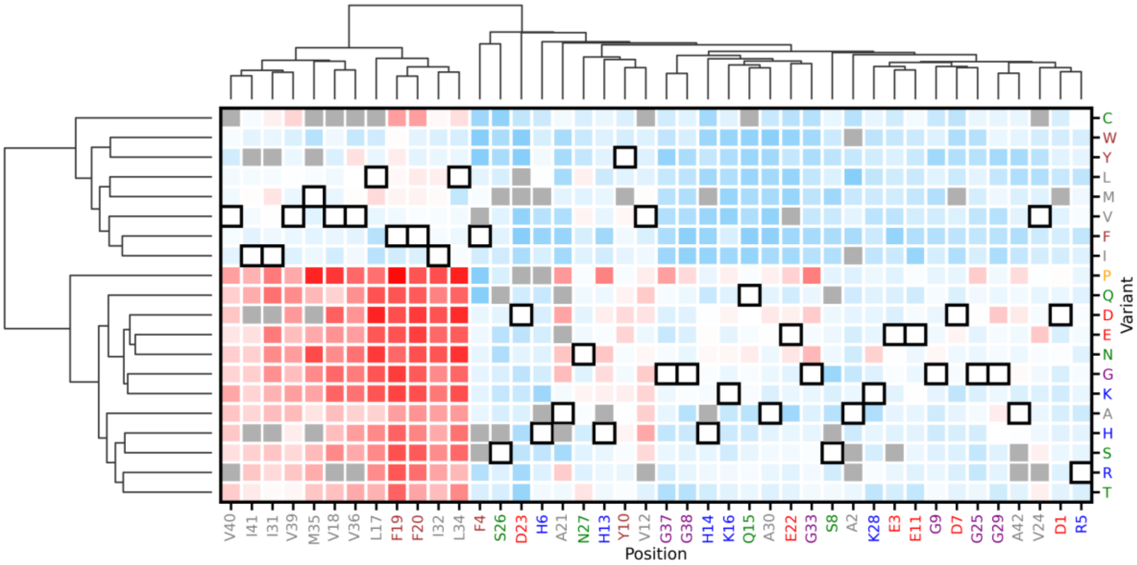

Supplementary Figure 5: Heatmap of $A\beta_{42}$ variant fitness clustered by both variant effect (vertical axis) and primary sequence context of the substitution. Wild-type amino acids are highlighted with a black box. Absent variants are coloured grey. Hierarchical clustering was applied to both axes, according to the dendrogram. To enable this, missing values were estimated as the average fitness score for a variant of that amino acid.

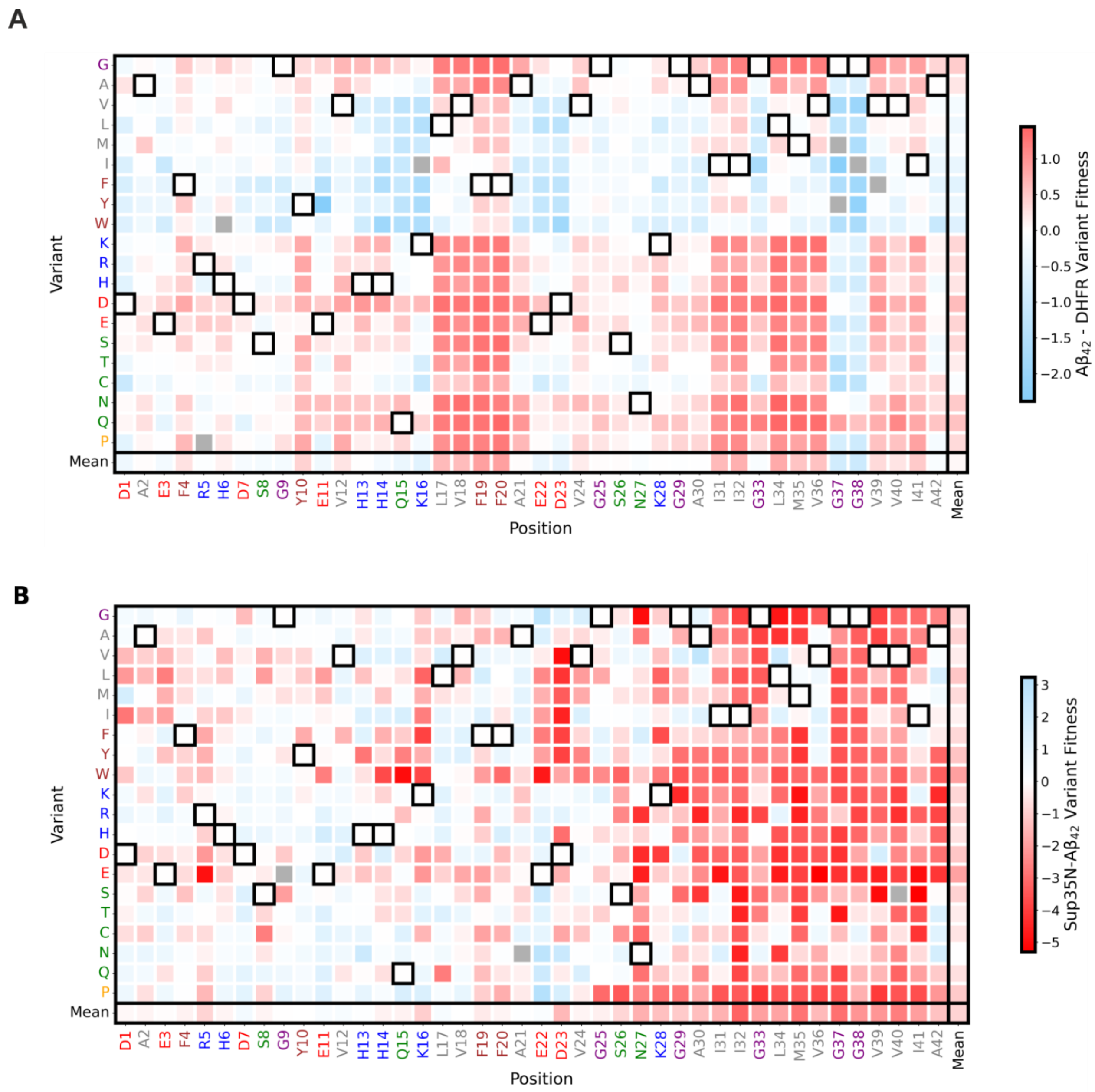

Supplementary Figure 6: **DMS data obtained by others for Aβ$_{42}$ in the yeast cytoplasm. (A)** Heatmap of variant fitness scores for single residue mutants obtained using Aβ$_{42}$-DHFR (17) and **(B)** Sup35N-Aβ$_{42}$ (18). The horizontal axis displays the wild-type (WT) amino acid sequence. The vertical axis shows the variant amino acid introduced at each position in the sequence. Wild-type amino acids are highlighted with a black box. Absent variants are coloured grey. The mean fitness value of each type of amino acid introduced is shown as the right-hand column, and the mean value for all residues introduced at each site in the sequence is shown as the bottom row in both plots.

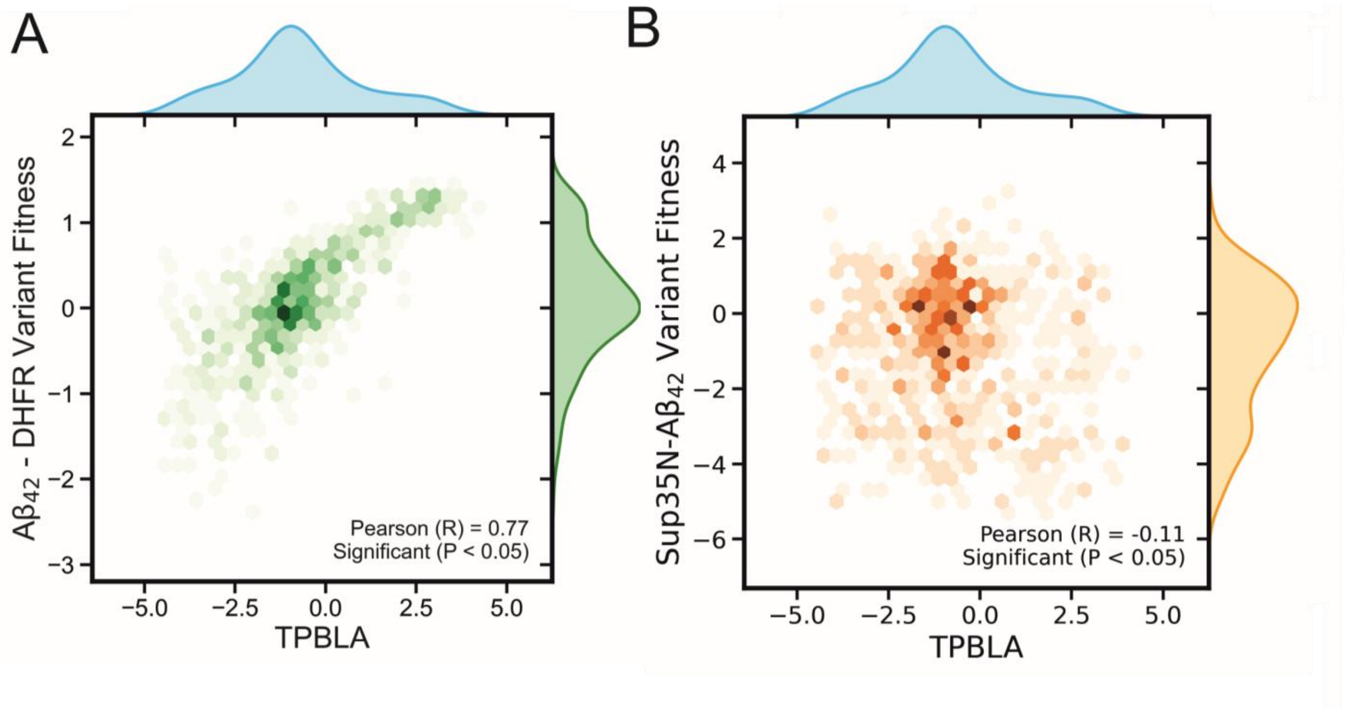

Supplementary Figure 7: **Comparison between TPBLA fitness scores and those obtained for Aβ_{42}-DHFR and Sup35N-Aβ_{42} in the yeast cytoplasm**. (**A**) Density plot comparing TPBLA variant fitness scores with those from the Aβ_{42}-DHFR dataset (R = 0.77 P<0.05) (17). (**B**) Density plot comparing TPBLA variant fitness scores with those from the Sup35N-Aβ_{42} dataset (R = -0.1 P<0.05) (18).

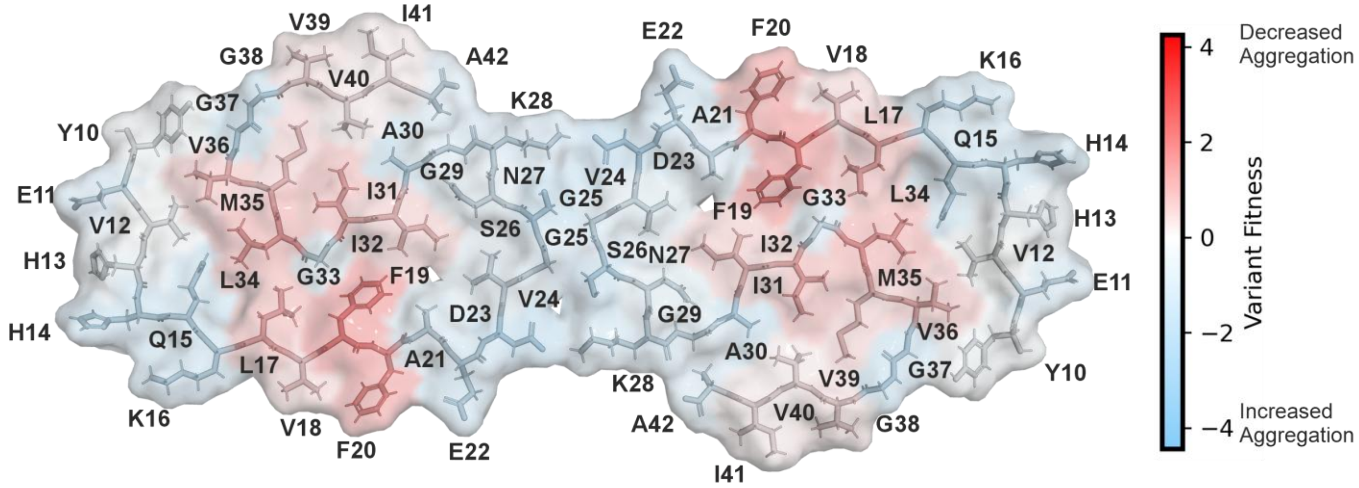

Supplementary Figure 8: Stabilising regions in amyloid structures correlate with TPBLA fitness scores. Residues in the cryo-EM structure of $A\beta_{42}$ fibrils (PDB:8OLH(19)), which achieved the median agreement between FoldX-determined fibril stability and TPBLA variant fitness score (Figure 4) are coloured according to the average variant fitness score for all variants sampled at each residue position. This highlights areas that stabilise the structure (red) and those that are points of frustration (blue).

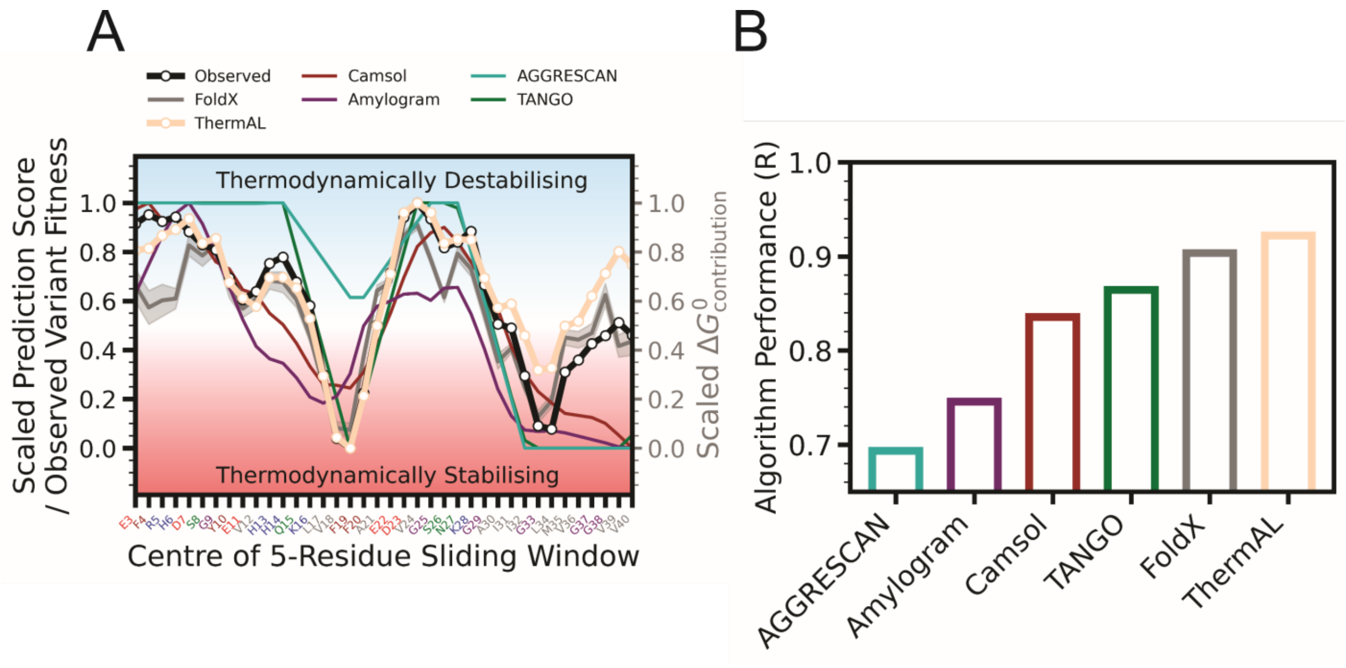

Supplementary Figure 9: Comparing the correlation of FoldX-derived $\Delta G^{0}_{contribution}$ and algorithms used to predict amyloid propensity with observed variant fitness (A) Shown is the per-residue $\Delta G^{0}_{contribution}$ computed by FoldX for all Aβ_{40} and Aβ_{42} (grey) structures over a 5-residue sliding window averaged over all structures (20). Additionally shown are the results of AGGRESCAN (21), TANGO (22), Amylogram (23), Camsol (24) and ThermAL averaged over a 5-residue sliding window, coloured according to the legend. Finally in black is the average observed variant fitness scores from TPBLA averaged over a 5-residue sliding window. (**B**) Computed Pearson between all datasets shown on the left and average observed variant fitness scores from TPBLA averaged over a 5-residue sliding window.

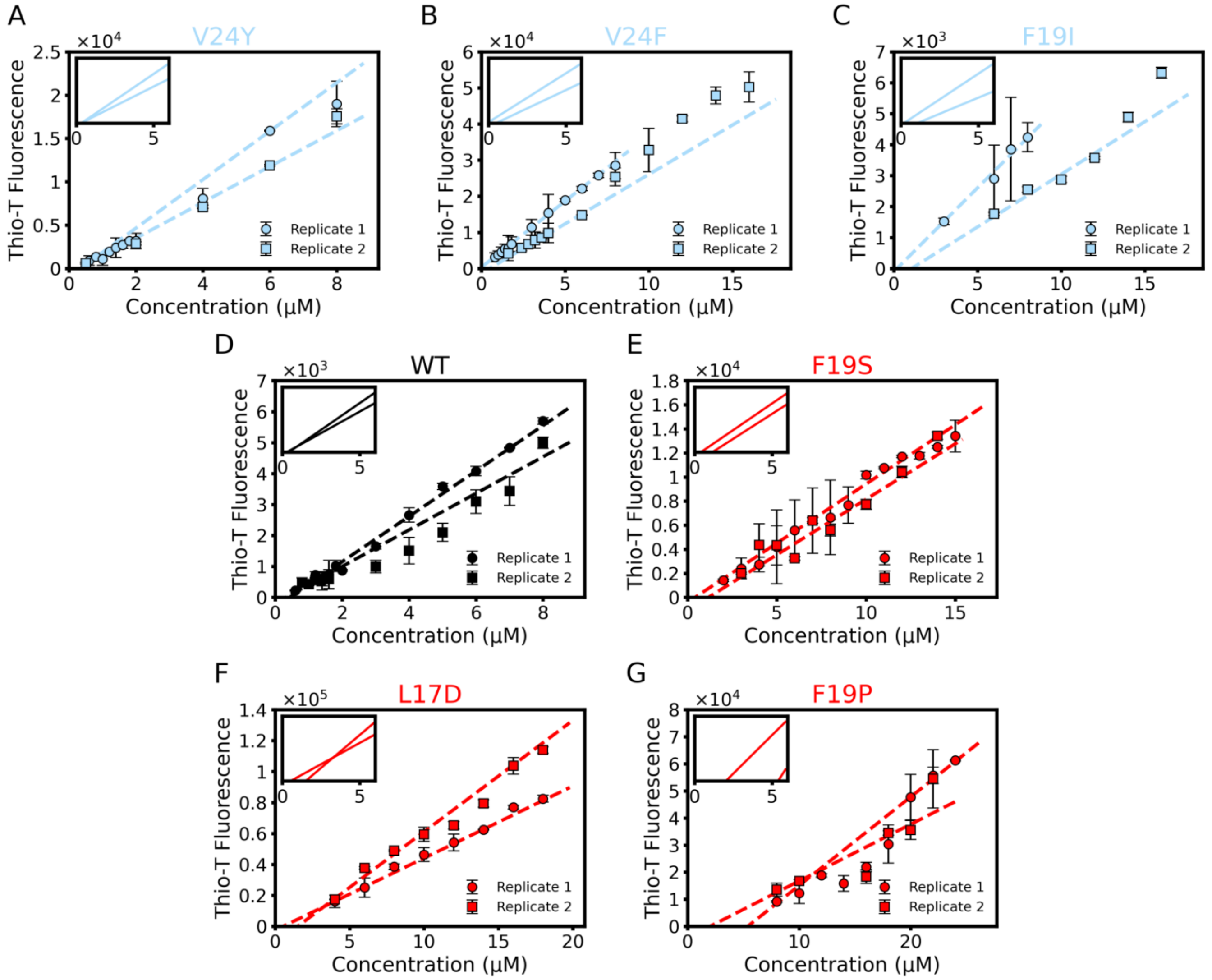

Supplementary Figure 10: Determination of C_{crit} using endpoint ThioT fluorescence intensity over a range of initial Aβ_{42} monomer concentrations for the variants **(A)** V24Y, (**B)** V24F, **(C)** F19I, **(D)** wild-type**, (E)** F19S, **(F)** L17D and **(G)** F19P (Methods). Extrapolation of the x-axis intercept enables estimation of the C_{crit} for amyloid formation. Inset shows the best fit lines for biological repeats magnified to the same scale to facilitate direct comparison.

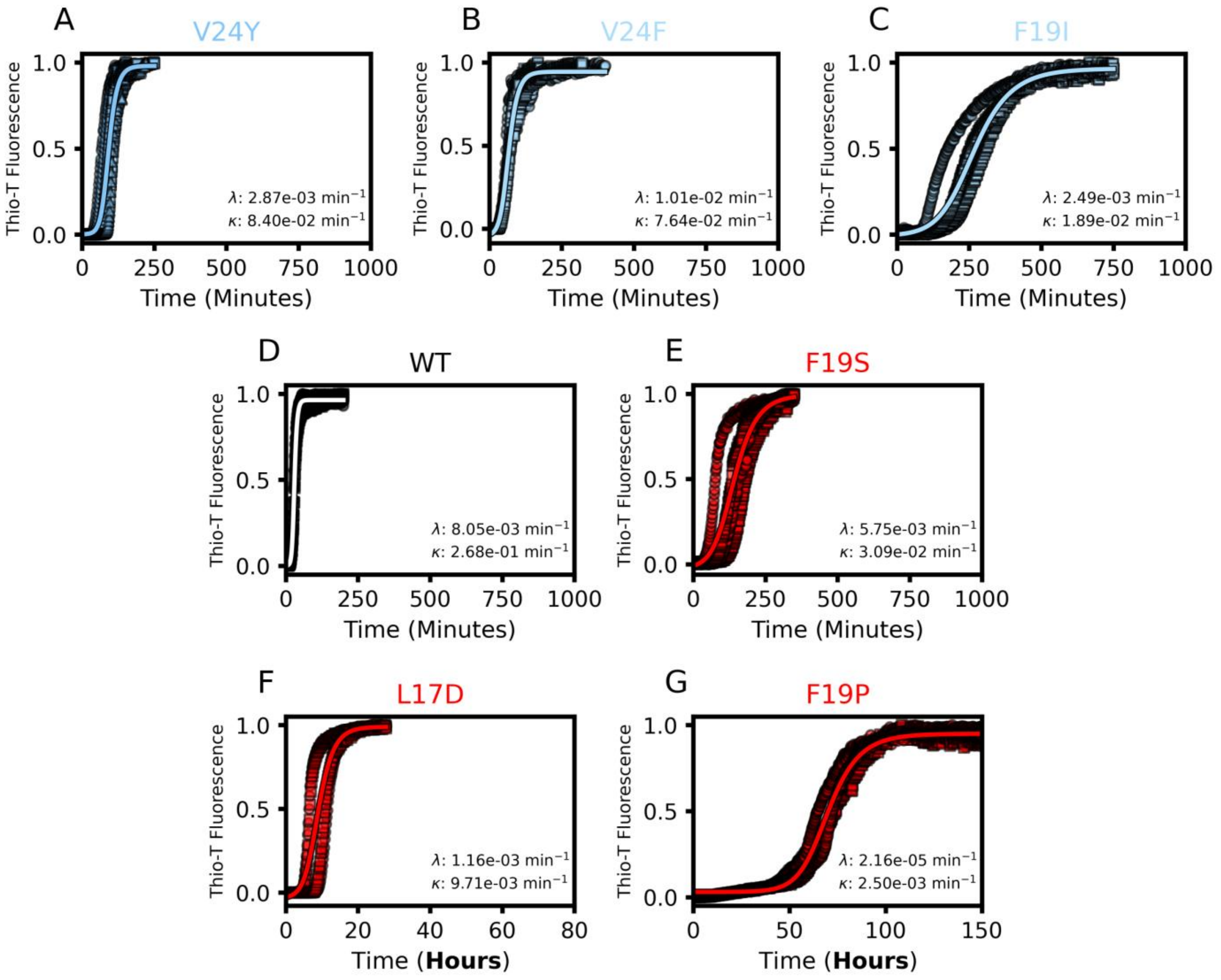

Supplementary Figure 11: **Amyloid formation kinetics for a panel of Aβ_{42} variants.** Amyloid formation kinetics were determined for the variants **(A)** V24Y, (**B)** V24F, **(C)** F19I, **(D)** wild-type**, (E)** F19S, **(F)** L17D and **(G)** F19P monitored by ThioT fluorescence using an initial monomer concentration of 8 μM. Macroscopic rate constants κ and λ were extracted by fitting the data as described in methods and from citation (10).

.

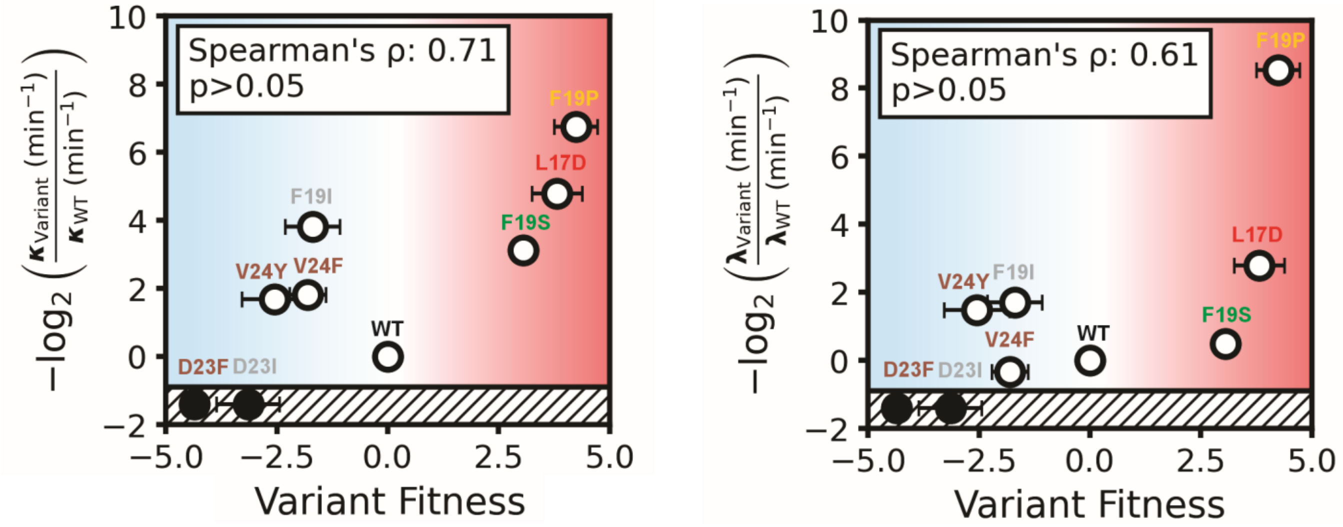

Supplementary Figure 12: **Correlation of κ and λ with variant fitness scores for a panel of purified variants (D23F, D23I, V24Y, V24F, F19I, wild-type, F19S, L17D and F19P).** Variants D23F and D23I could not be purified in monomeric form and are shown in the hatched region of the phase diagram in black. κ and λ were derived by fitting the kinetic profile of amyloid formation monitored using ThioT fluorescence using an 8 µM initial monomer concentration (Methods). Annotated are the variant identities coloured by the identity of the introduced amino acid class. No correlation was observed between TPBLA variant fitness scores and **(A)** $\log_2(\kappa_{Variant})/\log_2(\kappa_{WT})$ ($\rho=0.71$, $p>0.05$) or **(B)** $\log_2((\lambda_{Variant})/\log_2(\lambda_{WT}))$ ($\rho=0.61$, $p>0.05$).

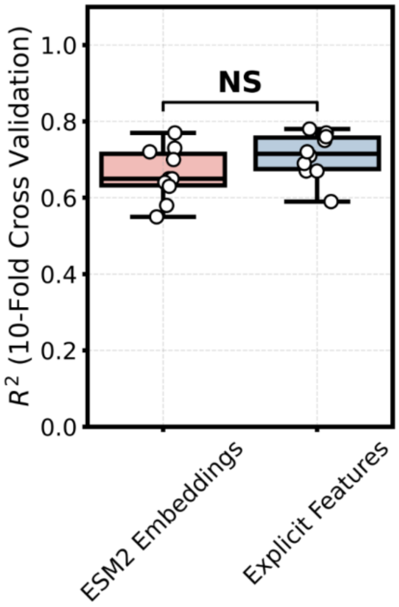

Supplementary Figure 13: Comparison of Model Performance (R^2) for Random Forest Models trained on the TPBLA βLa-Aβ$_{42}$ dataset. Models were trained using either sequence embeddings extracted from ESM2 or explicit sequence-derived empirical features including one-hot encoded amino acid identities, dipeptide composition, and additional sequence-derived features, as indicated. "NS" denotes a non-significant difference between the two groups. Scatter points represent R^2 values obtained from each fold in the 10-fold cross-validation, and error bars indicate the variability within these folds.

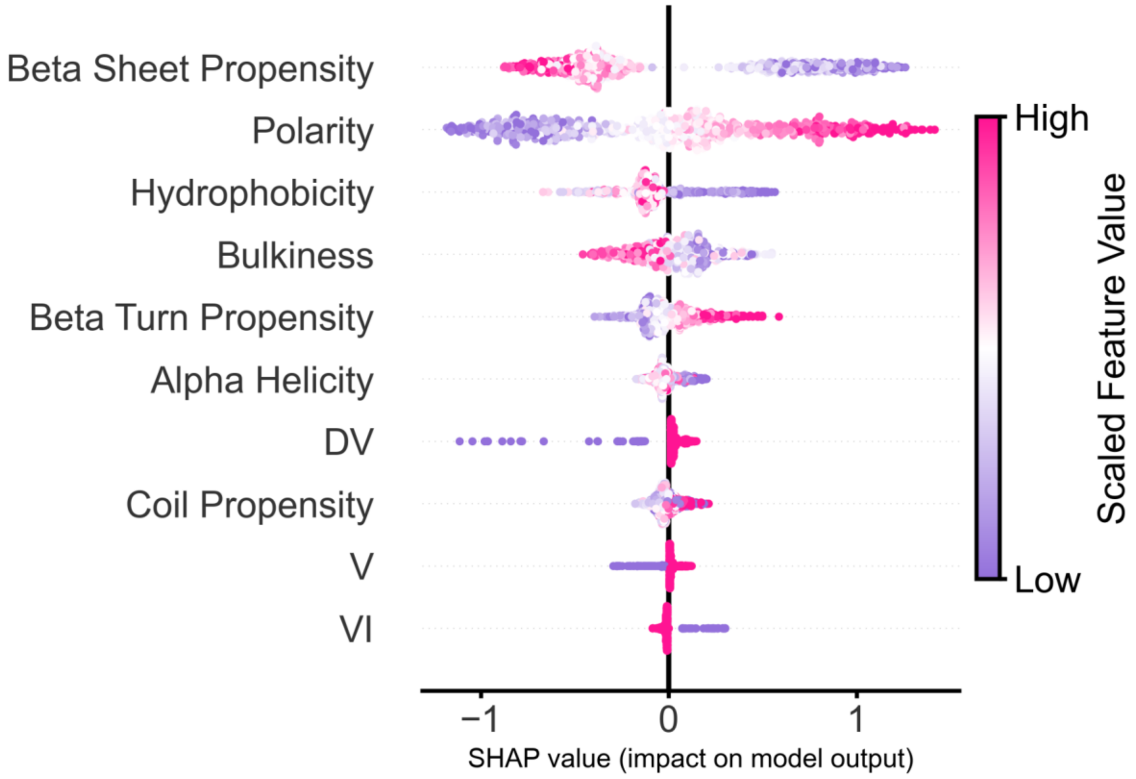

Supplementary Figure 14: SHAP values derived from the β-lactamase-trained Aβ_{42} Random Forest (ThermAL) for features with the highest SHAP values. DV and VI are dipeptides content and V is Valine content. The colours represent the magnitude of the corresponding scaled feature values.

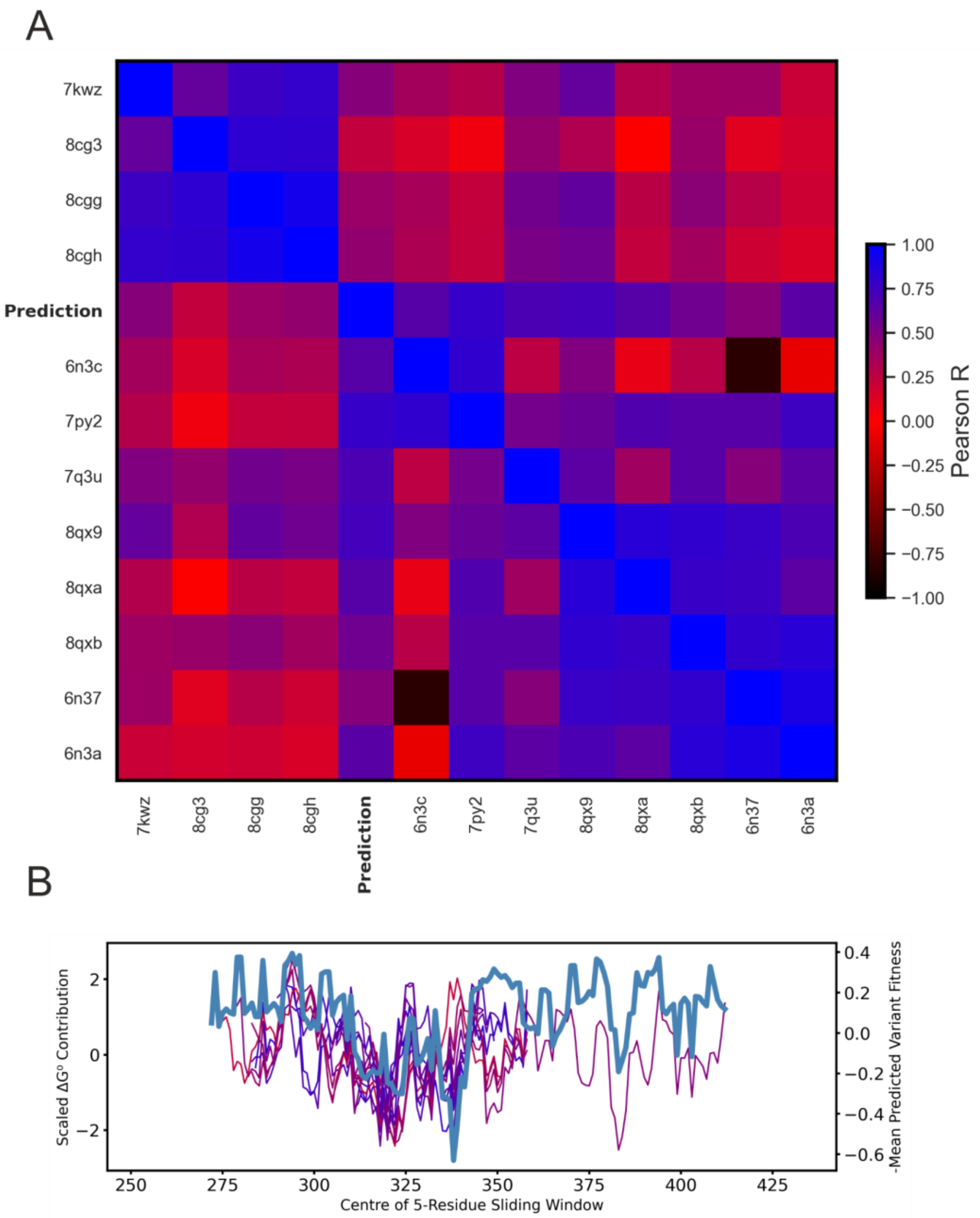

Supplementary Figure 15: **Inter-relatedness of TDP-43 fibril structures by their FoldX-determined fibril stability and βla-Aβ$_{42}$-trained model prediction (ThermAL)**. **Upper panel**: Hierarchically clustered Pearson correlations between regions stabilising TDP-43 fibril structures and regions predicted to stabilise the amyloid fold by the βla-Aβ$_{42}$-trained model. **Lower Panel**: Superimposition of FoldX-calculated scaled ΔG° per residue contribution of TDP-43 fibril structures coloured by their agreement with the βla-Aβ$_{42}$-trained model, which is shown as a thick steel blue line. The predictions based on the βla-Aβ$_{42}$ dataset agree well with structures 7PY2 (25) (Pearson R= 0.79) and 8QX9 (26) (Pearson R= 0.73), 7Q3U (27) (Pearson R= 0.70), however, less agreement is observed with other structures such as 8CGG (28) (Pearson R= 0.39), 8CG3 (28) (Pearson R= 0.24) and 8CGH (28) (Pearson R= 0.42).

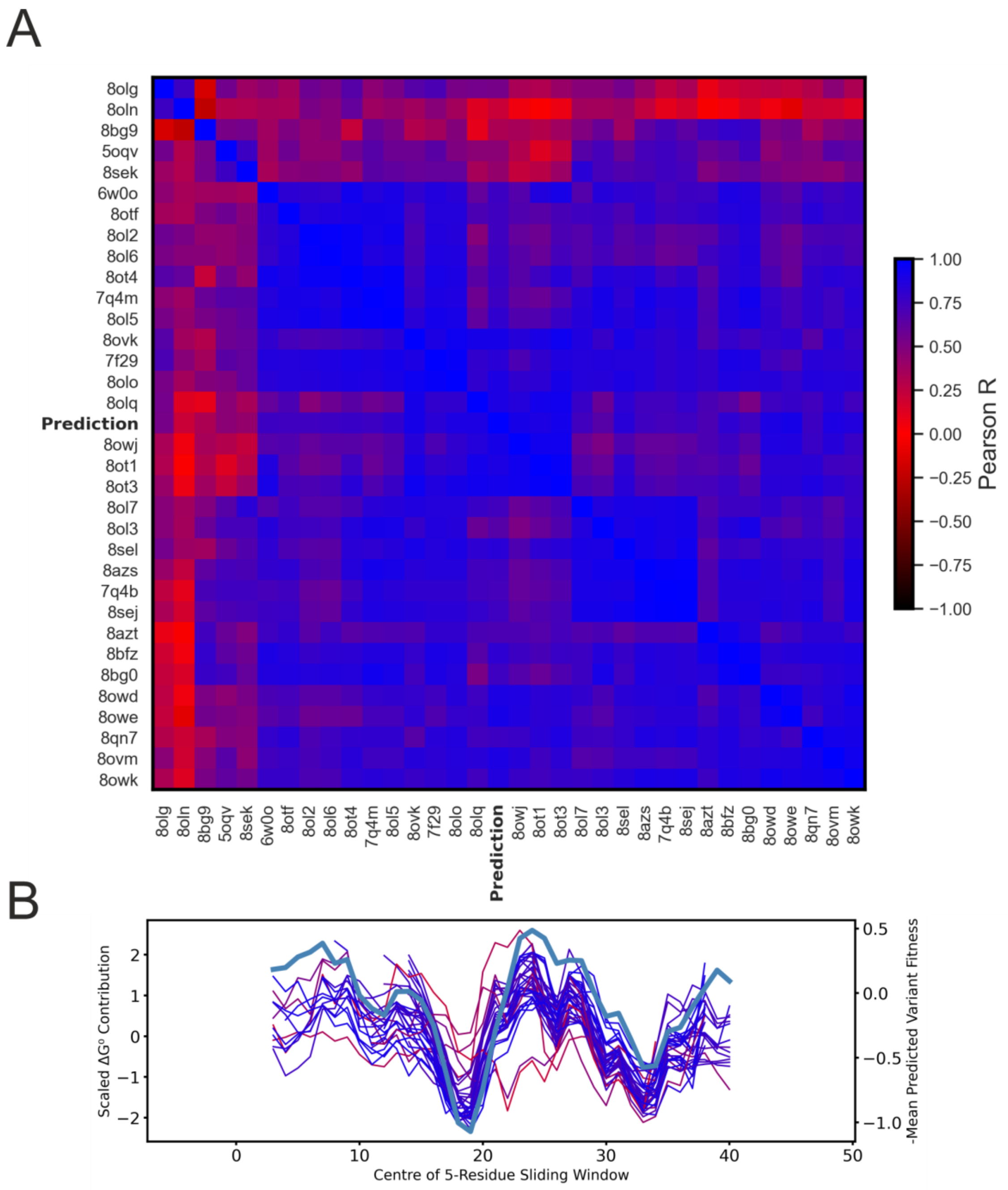

Supplementary Figure 16: **Inter-relatedness of Aβ_{42} fibrils structures by their FoldX-determined fibril stability and βla-Aβ_{42}-trained model prediction (ThermAL)**. **Upper panel**: Hierarchically clustered Pearson correlations between regions stabilising Aβ_{42} fibril structures and regions predicted to stabilise their amyloid folds by the βla-Aβ_{42}-trained model. **Lower Panel**: Superimposition of Aβ_{42} fibril structures based on scaled ΔG° contribution coloured by their agreement with the βla-Aβ_{42}-trained model, which is shown as a thick steel blue line.

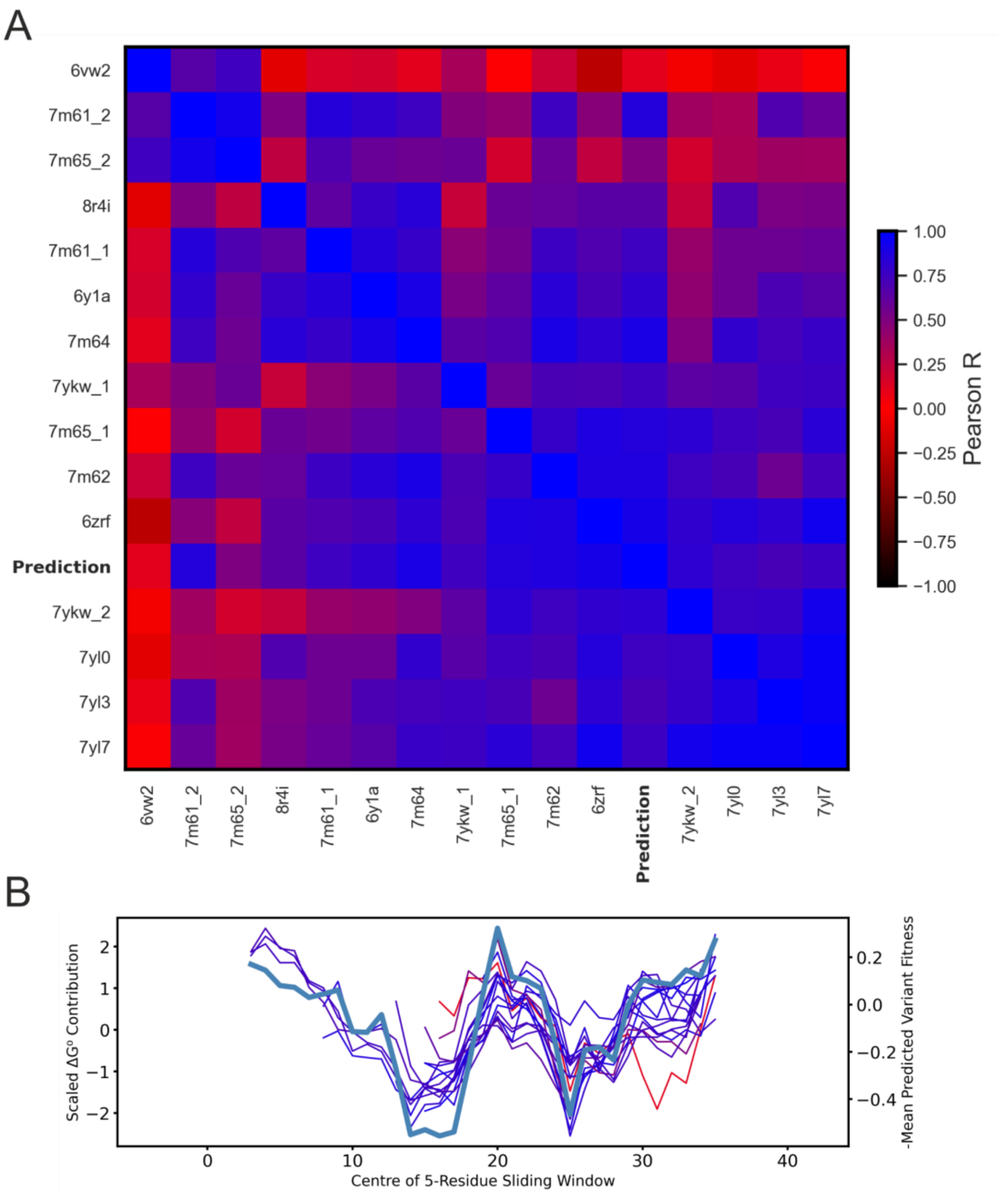

Supplementary Figure 17: **Inter-relatedness of hIAPP fibrils structures by their FoldX-determined fibril stability and β-lactamase Aβ$_{42}$-trained model prediction (ThermAL)**. **Upper panel**: Hierarchically clustered Pearson correlations between regions stabilising human IAPP fibril structures and regions predicted to stabilise the amyloid by the β-lactamase Aβ$_{42}$-trained model. **Lower Panel**: Superimposition of human IAPP fibril structures based on scaled ΔG° contribution coloured by their agreement with the βla-Aβ$_{42}$-trained model, which is shown as a thick steel blue line.

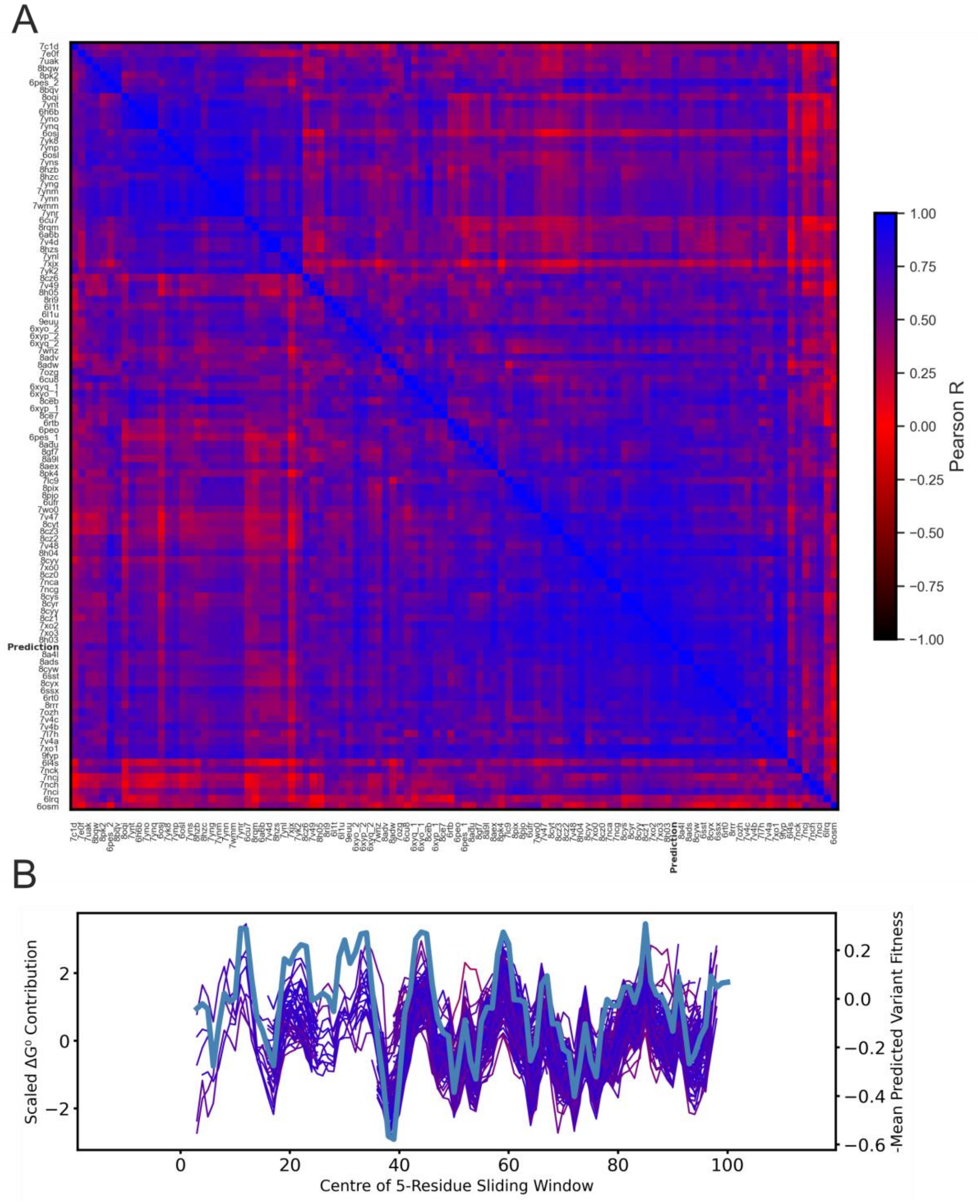

Supplementary Figure 18: **Inter-relatedness of α-synuclein fibrils structures by their FoldX-determined fibril stability and βla-Aβ$_{42}$-trained model prediction (ThermAL)**. **Upper panel**: Hierarchically clustered Pearson correlation between regions stabilising human α-synuclein fibril structures and regions predicted to stabilise the amyloid by the βla-Aβ$_{42}$-trained model. **Lower Panel**: Superimposition of α-synuclein fibril structures based on scaled ΔG° contribution coloured by their agreement with the βla-Aβ$_{42}$-trained model, which is shown as a thick steel blue line.

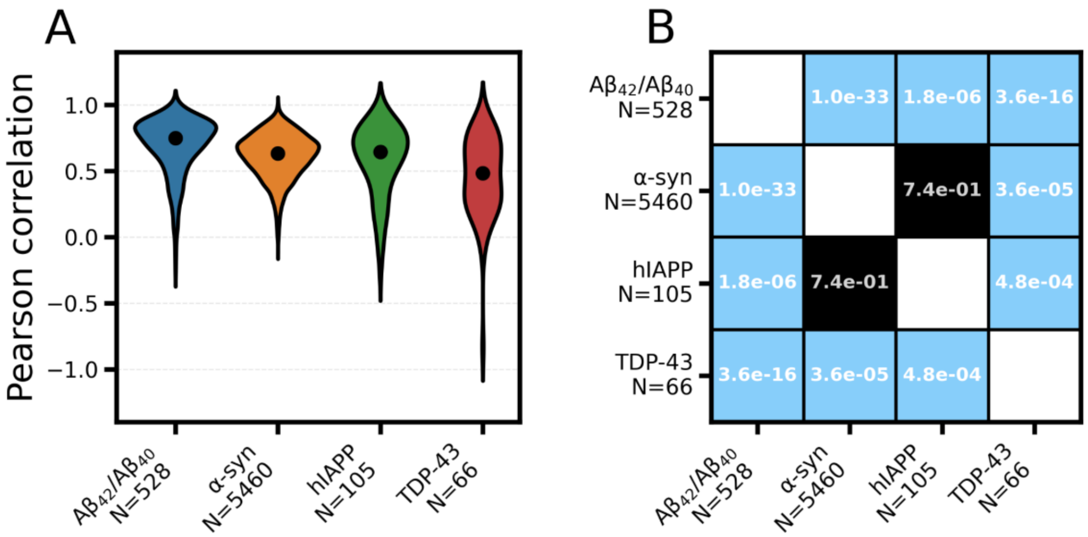

Supplementary Figure 19: (A) Violin plots of intra-protein relatedness for four amyloid-forming proteins: $A\beta_{42}/A\beta_{40}$, α-synuclein, hIAPP, and TDP-43. For each protein, Pearson correlation coefficients were calculated between every pair of fibril structures as shown in panel A of Figures 14-17. Each violin represents the full distribution of these pairwise correlations. The black dot inside each violin indicates the median correlation value. The number of pairwise comparisons (N) is displayed below each x-axis label. **(B)** Heatmap of pairwise statistical comparisons between the relatedness distributions shown in panel (A). Following a significant Kruskal–Wallis test, Dunn's test with false-discovery-rate correction was used to assess whether two proteins exhibit significantly different spreads of correlation. Each non-diagonal cell reports the p-value for that comparison. Blue cells ($p < 0.05$) denote a significant difference in distribution; black cells ($p \geq 0.05$) denote no significant difference. White diagonal cells indicate self-comparisons and are not tested. All proteins intra-protein relatedness were significant from other proteins with the exception of α-synuclein and hIAPP. The overall lower correlation distribution for TDP-43, together with its significant Dunn's test results, indicates that TDP-43 scaled ΔG° contributions vary more widely across fibril structures than those of the other protein families, reflecting greater heterogeneity in the TDP-43 dataset.

Supplementary References

1. Bushnell B, Rood J, Singer E. BBMerge – accurate paired shotgun read merging via overlap. PLoS One 12, e0185056 (2017).

2. Martin M. Cutadapt removes adapter sequences from high-throughput sequencing reads. EMBnet.journal 17, 10–12 (2011).

3. Langmead B, Trapnell C, Pop M, Salzberg SL. Ultrafast and memory-efficient alignment of short DNA sequences to the human genome. Genome Biol. 10, R25 (2009).

4. Radusky LG, Serrano L. pyFoldX: enabling biomolecular analysis and engineering along structural ensembles. Bioinformatics 38, 2353–2355 (2022).

5. Kapust RB, Tözsér J, Copeland TD, Waugh DS. The P1′ specificity of tobacco etch virus protease. Biochem. Biophys. Res. Commun. 294, 949–955 (2002).

6. Raran-Kurussi S, Cherry S, Zhang D, Waugh DS. Removal of affinity tags with TEV protease. Methods Mol. Biol. 1586, 221–230 (2017).

7. Abelein A, Chen G, Kitoka K, Aleksis R, Oleskovs F, Sarr M, et al. High-yield production of amyloid-β peptide enabled by a customized spider silk domain. Sci. Rep. 10, 235 (2020).

8. Zhong X, Kumar R, Wang Y, Biverstål H, Jegerschöld CI, Koeck PJB, Johansson J, Abelein A, Chen G. Amyloid Fibril Formation of Arctic Amyloid-β 1-42 Peptide is Efficiently Inhibited by the BRICHOS Domain. ACS Chem Biol. 17(8), 2201–2211 (2022)

9. Virtanen P, Gommers R, Oliphant TE, Haberland M, Reddy T, Cournapeau D, et al. SciPy 1.0: fundamental algorithms for scientific computing in Python. Nat. Methods 17, 261–272 (2020).

10. Dear AJ, Meisl G, Michaels TCT, Zimmermann MR, Linse S, Knowles TPJ. The catalytic nature of protein aggregation. J. Chem. Phys. 152, 045101 (2020).

11. Chen Z, Zhao P, Li F, Leier A, Marquez-Lago TT, Wang Y, et al. iFeature: a Python package and web server for feature extraction and selection from protein and peptide sequences. Bioinformatics 34, 2499–2502 (2018).

12. Wilkins MR, Gasteiger E, Bairoch A, Sanchez JC, Williams KL, Appel RD, et al. Protein identification and analysis tools in the ExPASy server. Methods Mol. Biol. 112, 531–552 (1999).

13. Gasteiger E, Hoogland C, Gattiker A, Duvaud S, Wilkins MR, Appel RD, et al. Protein identification and analysis tools on the ExPASy server. In: Walker JM, Ed. The Proteomics Protocols Handbook. (Humana Press, 2005), pp. 571–607.

14. Pedregosa F, Varoquaux G, Gramfort A, Michel V, Thirion B, Grisel O, et al. Scikit-learn: machine learning in Python. J. Mach. Learn. Res. 12, 2825–2830 (2011).

15. Harun R, Lu J, Kassir N, Zhang W. Machine learning–based quantification of patient factors impacting remission in patients with ulcerative colitis: insights from etrolizumab phase III clinical trials. Clin. Pharmacol. Ther. 115, 815–824 (2024).

16. Ponce-Bobadilla AV, Schmitt V, Maier CS, Mensing S, Stodtmann S. Practical guide to SHAP analysis: explaining supervised machine learning model predictions in drug development. Clin. Transl. Sci. 17, e70056 (2024).

17. Gray VE, Sitko K, Kameni FZN, Williamson M, Stephany JJ, Hasle N, et al. Elucidating the molecular determinants of Aβ aggregation with deep mutational scanning. G3 (Bethesda) 9, 3683–3689 (2019).

18. Seuma M, Lehner B, Bolognesi B. An atlas of amyloid aggregation: the impact of substitutions, insertions, deletions and truncations on amyloid beta fibril nucleation. Nat. Commun. 13, 7084 (2022).

19. Zielinski M, Peralta Reyes FS, Gremer L, Schemmert S, Frieg B, Schäfer LU, et al. Cryo-EM of Aβ fibrils from mouse models find tg-APPArcSwe fibrils resemble those found in patients with sporadic Alzheimer’s disease. Nat. Neurosci. 26, 2073–2080 (2023).

20. Schymkowitz J, Borg J, Stricher F, Nys R, Rousseau F, Serrano L. The FoldX web server: an online force field. Nucleic Acids Res. 33 (Suppl 2), W382–W388 (2005).

21. Conchillo-Solé O, de Groot NS, Avilés FX, Vendrell J, Daura X, Ventura S. AGGRESCAN: a server for the prediction and evaluation of ‘hot spots’ of aggregation in polypeptides. BMC Bioinformatics 8, 65 (2007).

22. Fernández-Escamilla AM, Rousseau F, Schymkowitz J, Serrano L. Prediction of sequence-dependent and mutational effects on the aggregation of peptides and proteins. Nat. Biotechnol. 22, 1302–1306 (2004).

23. Burdukiewicz M, Sobczyk P, Rödiger S, Duda-Madej A, Mackiewicz P, Kotulska M. Amyloidogenic motifs revealed by n-gram analysis. Sci. Rep. 7, 12961 (2017).

24. Sormanni P, Vendruscolo M. Protein solubility predictions using the CamSol method in the study of protein homeostasis. Cold Spring Harb. Perspect. Biol. 11, a033845 (2019).

25. Arseni D, Hasegawa M, Murzin AG, Kametani F, Arai M, Yoshida M, et al. Structure of pathological TDP-43 filaments from ALS with FTLD. Nature 601, 139–143 (2022).

26. Sharma K, Stockert F, Shenoy J, Berbon M, Abdul-Shukkoor MB, Habenstein B, et al. Cryo-EM observation of the amyloid key structure of polymorphic TDP-43 amyloid fibrils. Nat. Commun. 15, 486 (2024).

27. Kumar ST, Nazarov S, Porta S, Maharjan N, Cendrowska U, Kabani M, et al. Seeding the aggregation of TDP-43 requires post-fibrillization proteolytic cleavage. Nat. Neurosci. 26, 983–986 (2023).

28. Arseni D, Chen R, Murzin AG, Peak-Chew SY, Garringer HJ, Newell KL, et al. TDP-43 forms amyloid filaments with a distinct fold in type A FTLD-TDP. Nature 620, 898–903 (2023).
